# Genetic Association for Renal Traits among Participants of African Ancestry Reveals New Loci for Renal Function

**DOI:** 10.1371/journal.pgen.1002264

**Published:** 2011-09-08

**Authors:** Ching-Ti Liu, Maija K. Garnaas, Adrienne Tin, Anna Kottgen, Nora Franceschini, Carmen A. Peralta, Ian H. de Boer, Xiaoning Lu, Elizabeth Atkinson, Jingzhong Ding, Michael Nalls, Daniel Shriner, Josef Coresh, Abdullah Kutlar, Kirsten Bibbins-Domingo, David Siscovick, Ermeg Akylbekova, Sharon Wyatt, Brad Astor, Josef Mychaleckjy, Man Li, Muredach P. Reilly, Raymond R. Townsend, Adebowale Adeyemo, Alan B. Zonderman, Mariza de Andrade, Stephen T. Turner, Thomas H. Mosley, Tamara B. Harris, The CKDGen Consortium, Charles N. Rotimi, Yongmei Liu, Sharon L. R. Kardia, Michele K. Evans, Michael G. Shlipak, Holly Kramer, Michael F. Flessner, Albert W. Dreisbach, Wolfram Goessling, L. Adrienne Cupples, W. Linda Kao, Caroline S. Fox

**Affiliations:** 1 Department of Biostatistics, Boston University School of Public Health, Boston, Massachusetts, United States of America; 2 Division of Genetics, Department of Medicine, Brigham and Women's Hospital and Harvard Medical School, Boston, Massachusetts, United States of America; 3 Harvard Stem Cell Institute, Harvard University, Cambridge, Massachusetts, United States of America; 4 Department of Epidemiology, Johns Hopkins University, Baltimore, Maryland, United States of America; 5 Renal Division, University Hospital of Freiburg, Freiburg, Germany; 6 Department of Epidemiology, University of North Carolina at Chapel Hill, Chapel Hill, North Carolina, United States of America; 7 Division of Nephrology, University of California San Francisco Medical School and San Francisco VA Medical Center, San Francisco, California, United States of America; 8 Division of Nephrology and Kidney Research Institute, University of Washington, Seattle, Washington, United States of America; 9 Division of Biomedical Statistics and Informatics, Mayo Clinic, Rochester, Minnesota, United States of America; 10 Gerontology and Geriatric Medicine, J. Paul Sticht Center on Aging, Wake Forest University Health Sciences, Winston-Salem, North Carolina, United States of America; 11 Laboratory of Neurogenetics, National Institute of Aging, National Institutes of Health, Bethesda, Maryland, United States of America; 12 Center for Research on Genomics and Global Health, National Human Genome Research Institute, Bethesda, Maryland, United States of America; 13 Welch Center for Prevention, Epidemiology, and Clinical Research, Johns Hopkins University, Baltimore, Maryland, United States of America; 14 Medical College of Georgia, Augusta, Georgia, United States of America; 15 University of California San Francisco, San Francisco, California, United States of America; 16 Cardiovascular Health Research Unit, Departments of Epidemiology and Medicine, University of Washington, Seattle, Washington, United States of America; 17 Jackson State University, Jackson, Mississippi, United States of America; 18 School of Nursing, University of Mississippi Medical Center, Jackson, Mississippi, United States of America; 19 Department of Epidemiology, Bloomberg School of Public Health, Baltimore, Maryland, United States of America; 20 Center for Public Health Genomics, Charlottesville, Virginia, United States of America; 21 Department of Epidemiology, Johns Hopkins University, Baltimore, Maryland, United States of America; 22 Cardiovascular Institute, University of Pennsylvania, Philadelphia, Pennsylvania, United States of America; 23 Renal Electrolyte and Hypertension Division, University of Pennsylvania, Philadelphia, Pennsylvania, United States of America; 24 Laboratory of Personality and Cognition, National Institute on Aging, National Institutes of Health, Baltimore, Maryland, United States of America; 25 Department of Internal Medicine, Division of Nephrology and Hypertension, Mayo Clinic, Rochester, Minnesota, United States of America; 26 Division of Geriatrics, Department of Medicine, University of Mississippi Medical Center, Jackson, Mississippi, United States of America; 27 Laboratory of Epidemiology, Demography, and Biometry, National Institute on Aging, National Institutes of Health, Bethesda, Maryland, United States of America; 28 Department of Epidemiology and Prevention, Division of Public Health Sciences, Wake Forest University, Winston-Salem, North Carolina, United States of America; 29 Department of Epidemiology, University of Michigan School of Public Health, Ann Arbor, Michigan, United States of America; 30 Health Disparities Research Section, Clinical Research Branch, National Institute on Aging, National Institutes of Health, Baltimore, Maryland, United States of America; 31 General Internal Medicine, University of California San Francisco, San Francisco, California, United States of America; 32 Loyola University, Maywood, Illinois, United States of America; 33 Division of Kidney, Urologic, and Hematologic Diseases, National Institute of Diabetes and Digestive and Kidney Diseases, National Institutes of Health, Bethesda, Maryland, United States of America; 34 University of Mississippi Division of Nephrology, University of Mississippi, Jackson, Mississippi, United States of America; 35 Divisions of Genetics and Gastroenterology, Department of Medicine, Brigham and Women's Hospital and Harvard Medical School, Boston, Massachusetts, United States of America; 36 Department of Biostatistics, Boston University School of Public Health and National Heart, Blood, and Lung Institute's Framingham Heart Study, Boston, Massachusetts, United States of America; 37 National Heart, Blood, and Lung Institute's Framingham Heart Study and the Center for Population Studies, Framingham, Massachusetts, United States of America; 38 Division of Endocrinology, Brigham and Women's Hospital and Harvard Medical School, Boston, Massachusetts, United States of America; University of Alabama, Birmingham, United States of America

## Abstract

Chronic kidney disease (CKD) is an increasing global public health concern, particularly among populations of African ancestry. We performed an interrogation of known renal loci, genome-wide association (GWA), and IBC candidate-gene SNP association analyses in African Americans from the CARe Renal Consortium. In up to 8,110 participants, we performed meta-analyses of GWA and IBC array data for estimated glomerular filtration rate (eGFR), CKD (eGFR <60 mL/min/1.73 m^2^), urinary albumin-to-creatinine ratio (UACR), and microalbuminuria (UACR >30 mg/g) and interrogated the 250 kb flanking region around 24 SNPs previously identified in European Ancestry renal GWAS analyses. Findings were replicated in up to 4,358 African Americans. To assess function, individually identified genes were knocked down in zebrafish embryos by morpholino antisense oligonucleotides. Expression of kidney-specific genes was assessed by *in situ* hybridization, and glomerular filtration was evaluated by dextran clearance. Overall, 23 of 24 previously identified SNPs had direction-consistent associations with eGFR in African Americans, 2 of which achieved nominal significance (*UMOD*, *PIP5K1B*). Interrogation of the flanking regions uncovered 24 new index SNPs in African Americans, 12 of which were replicated (*UMOD*, *ANXA9*, *GCKR*, *TFDP2*, *DAB2*, *VEGFA*, *ATXN2*, *GATM*, *SLC22A2*, *TMEM60*, *SLC6A13*, and *BCAS3*). In addition, we identified 3 suggestive loci at *DOK6* (p-value = 5.3×10^−7^) and *FNDC1* (p-value = 3.0×10^−7^) for UACR, and *KCNQ1* with eGFR (p = 3.6×10^−6^). Morpholino knockdown of kcnq1 in the zebrafish resulted in abnormal kidney development and filtration capacity. We identified several SNPs in association with eGFR in African Ancestry individuals, as well as 3 suggestive loci for UACR and eGFR. Functional genetic studies support a role for kcnq1 in glomerular development in zebrafish.

## Author Summary

Chronic kidney disease (CKD) is an increasing global public health problem and disproportionately affects populations of African ancestry. Many studies have shown that genetic variants are associated with the development of CKD; however, similar studies are lacking in African ancestry populations. The CARe consortium consists of more than 8,000 individuals of African ancestry; genome-wide association analysis for renal-related phenotypes was conducted. In cross-ethnicity analyses, we found that 23 of 24 previously identified SNPs in European ancestry populations have the same effect direction in our samples of African ancestry. We also identified 3 suggestive genetic variants associated with measurement of kidney function. We then tested these genes in zebrafish knockdown models and demonstrated that kcnq1 is involved in kidney development in zebrafish. These results highlight the similarity of genetic variants across ethnicities and show that cross-species modeling in zebrafish is feasible for genes associated with chronic human disease.

## Introduction

Chronic kidney disease (CKD) affects approximately 15% of U.S adults [1]. Due in part to increasing rates of diabetes and obesity, the prevalence of CKD continues to rise [1]. Marked variability in the incidence of CKD suggests that factors other than diabetes and hypertension contribute to its etiology [2]. Recently, we identified 16 genomic loci associated with estimated glomerular filtration rate (eGFR), a primary measure of CKD, using genome-wide association studies (GWAS) in a combined sample of 67,093 European ancestry individuals from the CKDGen consortium [3], [4]. However, these loci only account for 1.4% of the eGFR variation, suggesting that additional loci remain to be identified [5].

African American ethnicity is a well-established risk factor for CKD, and rates of end-stage renal disease (ESRD) are up to 4-fold higher among African Americans as compared to European Americans [6]. Several prior studies, including the FIND consortium, have performed linkage analysis of diabetic ESRD [7]–[9]. Recent genome-wide admixture mapping studies identified genetic variation in the regions of *MYH9* and *APOL1* on chromosome 22 that may explain up to 70% of the differences in ESRD rates between European and African Americans [10]–[12]. While this finding has great implications for ESRD, recent evidence also suggests that African Americans progress faster from moderately decreased kidney function to ESRD, spending less time in the recognized earlier stages of CKD [13], [14]. The identification of additional risk factors for CKD, including genetic loci in association with eGFR, may help to advance our understanding of the underpinnings of CKD in African Americans.

Thus, the goal of this study was to uncover loci for kidney traits in African ancestry participants in the Candidate-gene Association Resource (CARe) Consortium. CARe is a consortium of 9 studies which form a combined population of approximately 40,000 African and European Americans genotyped on the IBC array [15] and approximately 8,000 African American participants genotyped using a GWAS platform. We aimed to interrogate known regions previously associated with eGFR in European Ancestry populations [3], [4], as well as to perform discovery analyses in African ancestry populations. In order to gain further insight into the functional implications of our associations, genes at or near loci of interest were knocked down by morpholino injection in zebrafish, and kidney gene expression and function were investigated using *in situ* hybridization and glomerular filtration assays.

## Results

Study sample characteristics are shown in Table 1 and Table S1. Overall, 7,382 participants of African ancestry had genome-wide SNP data and phenotype information on eGFR and CKD; and 5,569 had data on UACR and albuminuria. For the IBC chip analysis, 8,110 participants of African ancestry had data on eGFR and CKD and 5,995 had data on UACR and albuminuria.

**Table 1:** Study sample characteristics in the CARE Renal Consortium.*

Study	Sample Size eGFRcrea/UACR	European Ancestry§ %	Women %	Age (years)	eGFRcrea (ml/min/1.73 m^2^)	UACR (mg/g)§	MA (%)	CKD %
**CARE African Ancestry Stage 1 Participants**								
ARIC	2786/1936	15.3(10.7,22.1)	63.1	53.3	99.9	2.69(0.85,9.94)	16.5	3.7
CARDIA	821/754	16.7(12.2,23.2)	61.1	39.4	111	4.28(3.09,7.40)	9.4	0.9
CHS**	728/426	20.6(12.4,32.7)	62.8	72.9	81.4	10.95(5.00,26.90)	31.9	18.4
JHS	2135/1246	15.7(11.8,21.1)	60.8	50	101	6.00(4.00,12.00)	16.5	4.2
MESA	1640/1633	18.8(11.5,29.7)	54.8	62.2	86.5	5.50(3.10, 13.10)	16.4	8.6
Total	8110/5995							
**CARE European Ancestry**								
ARIC	9581/7687	NA	53.5	54.3	90.3	3.94(2.04,7.69)	9.3	3.5
CARDIA	1331/1242	NA	53.2	40.7	99.2	4.42(3.30,6.90)	4.5	0.5
CHS	3938/2073	NA	56.1	72.8	76.4	10.20(5.50,22.70)	26.4	21
FHS	6624/6208	NA	53.6	48.8	94	4.50(2.60,9.57)	9.1	3.9
MESA	2293/2287	NA	52.3	62.7	82.4	4.70(3.10,8.60)	9.5	9.7
Total	23767/19497							
**Stage 2: Replication**								
GENOA	1217/1228	12.6(7.2,18.9)	71.7	63.2	88	6.13 (2.97, 19.73)	22.9	13
HANDLS	989/629	16.1(11.2,22.0)	55	48.4	121	5.26(3.17,15.96)	20.3	5.3
Health ABC	1139/253	22.4 (12.2, 32.6)	57.2	73.4	76.3	19.3(5.7, 96.5)	47	17.1
HUFS	1013/NA	19.7(14.3,27.0)	58.8	48.3	104.3	NA	NA	4.9
Total	4358/2110							

Abbreviations: eGFRcrea: estimated glomerular filtration rate by serum creatinine, eGFRcys: estimated glomerular filtration rate by serum cystatin C, CKD: chronic kidney disease, HTN: hypertension, DM: diabetes mellitus, NA: not available.

* Sample characteristics based on the larger eGFR sample.

** CHS only has data on the IBC platform.

§ Median, 25/75^th^ percentile.

### Interrogation of Known Renal Function Loci in African American Participants

Genome-wide association analyses were conducted for eGFR, CKD, UACR, and albuminuria. Quantile-quantile and Manhattan plots are displayed in Figure S1 and Figure S2, respectively; lambdas ranged from 1.0 to 1.02.

We examined previously published loci in association with eGFR in participants of European Ancestry [3], [4], [16] in our African American GWAS (Table 2). In 23 of 24 SNPs, the directions of the beta coefficients for eGFR were identical (p-value = 1.4*10^−6^) in CKDGen and CARe (rs6420094 at *SLC34A1* was the only exception), even though only 2 of the SNPs achieved nominal significance (rs4293393 at the *UMOD* locus [p = 0.01] and rs4744712 at the *PIP5K1B* locus [p = 0.003]). We further interrogated the 250 kb flanking regions around each of these 24 SNP to identify the top SNP in CARe; statistical significance was determined based on a locus-specific Bonferroni correction (see statistical methods for more detail). Of the 24 SNPs with the lowest p-value in each of these regions identified in CARe African Americans, we were able to replicate 12 loci (*UMOD*, *ANXA9*, *GCKR*, *TFDP2*, *DAB2*, *VEGFA*, *ATXN2*, *GATM*, *SLC22A2*, *TMEM60*, *SLC6A13*, *and BCAS3*) in independent samples of 4358 participants (Table 3, Figure S3).

**Table 2:** Interrogation of known loci in EA in AA for the trait eGFRcrea; best SNP at each locus is shown below; SNP ID in bold from Kottgen et al, Nature Genetics 2009, and Nature Genetics 2010.

SNP ID in EA	Chr	Genes Within 60 kb	Coded Allele Frequency (allele)	Beta coefficient in EA relative to coded allele	Coded allele frequency for the lead SNPs in EA in AA (allele)	Beta coefficient in AA for the lead SNP in EA	p-value in AA for lead SNP in EA
**rs17319721**	4	SHROOM3	0.43 (A)	−0.013	0.22 (A)	−0.004	0.44
**rs10109414**	8	STC1	0.42 (T)	−0.008	0.34 (T)	−0.005	0.30
rs4293393*	16	UMOD	0.82 (A)	−0.016	0.81 (A)	−0.013	0.01
**rs267734**	1	ANXA9	0.8 (T)	−0.010	0.96 (T)	−0.016	0.13
**rs1260326**	2	GCKR	0.41 (T)	0.009	0.16 (T)	0.007	0.24
**rs13538**	2	ALMS1	0.77 (A)	−0.009	0.48 (A)	−0.003	0.57
**rs347685**	3	TFDP2	0.72 (A)	−0.009	0.75 (A)	−0.007	0.13
**rs11959928**	5	DAB2	0.44 (A)	−0.009	0.30 (A)	−0.002	0.75
**rs6420094**	5	SLC34A1	0.66 (A)	0.011	0.83 (A)	−0.002	0.83
**rs881858**	6	VEGFA	0.72 (A)	−0.011	0.36 (A)	−0.003	0.50
**rs10224210**	7	PRKAG2	0.73 (T)	0.010	0.92 (T)	0.019	0.06
**rs4744712**	9	PIP5K1B	0.39 (A)	−0.008	0.42 (A)	−0.015	0.0003
**rs653178**	12	ATXN2	0.51 (T)	0.003	0.92 (T)	0.001	0.87
**rs626277**	13	DACH1	0.60 (A)	−0.009	0.34 (A)	−0.003	0.50
**rs1394125**	15	UBE2Q2	0.35 (A)	−0.009	0.35 (A)	−0.007	0.15
**rs12460876**	19	SLC7A9	0.61 (T)	−0.008	0.72 (T)	−0.010	0.03
**rs10794720**	10	WDR37	0.08 (T)	−0.014	0.21 (T)	−0.007	0.18
**rs491567**	15	WDR72	0.78 (A)	−0.009	0.45 (A)	−0.001	0.87
**rs2453533**	15	SPATA5L1;GATM	0.38 (A)	−0.013	0.84 (A)	−0.008	0.18
**rs7422339**	2	CPS1	0.32 (A)	−0.009	0.32 (A)	−0.005	0.38
rs2279463**	6	SLC22A2	0.88 (A)	0.013	N/A	N/A	N/A
**rs6465825**	7	TMEM60	0.61 (T)	0.008	0.51 (T)	0.000	0.92
**rs10774021**	12	SLC6A13	0.64 (T)	−0.008	0.50 (T)	−0.005	0.23
**rs9895661**	17	BCAS3	0.81 (T)	0.011	0.53 (T)	0.008	0.05

* Lead SNP rs12917707 in EA not present in AA dataset; therefore used rs4293393, which is in perfect LD in EA (r2 = 1.0).

** rs2279463 not present in AA dataset.

**Table 3:** Interrogation of known loci in EA in AA for the trait eGFRcrea; best SNP at each locus is shown below; SNP ID in bold from Kottgen et al, Nature Genetics 2009, and Nature Genetics 2010.

SNP ID in EA	Chr	Best SNP ID in AA in the Region	Coded Allele Frequency in AA (allele)	Beta coefficient in AA for best SNP	P-value in AA	Number of independent (typed) SNPs interrogated in CARe AA	Bonferroni p-value threshhold (0.05/P)	Replication beta coefficient	Replication p-value	Stage 1+Stage 2 beta	Stage 1+Stage 2 p-value
**rs17319721**	4	rs4371638	0.19 (T)	−0.020	0.003	28	0.002	−0.002	0.42	−0.015	8.8E-03
**rs10109414**	8	rs192841	0.79 (A)	−0.016	0.007	29	0.002	0.012	0.91	−0.008	1.3E-01
**rs4293393**	16	rs4293393	0.81 (A)	−0.013	0.014	14	0.004	−0.017	0.02	−0.014	1.5E-03
**rs267734**	1	rs3738479	0.39 (A)	0.013	0.005	19	0.003	0.012	0.04	0.013	9.0E-04
**rs1260326**	2	rs13022873	0.81 (A)	0.013	0.017	17	0.003	0.007	0.21	0.011	1.3E-02
**rs13538**	2	rs7600291	0.58 (C)	0.011	0.011	19	0.003	−0.011	0.93	0.005	1.6E-01
**rs347685**	3	rs6781340	0.41 (T)	−0.014	0.002	21	0.002	−0.014	0.03	−0.014	2.1E-04
**rs11959928**	5	rs3822460	0.83 (T)	−0.013	0.020	11	0.005	−0.010	0.13	−0.012	1.0E-02
**rs6420094**	5	rs10463065	0.95 (C)	0.032	0.004	20	0.003	−0.001	0.52	0.023	1.6E-02
**rs881858**	6	rs1750571	0.07 (A)	0.023	0.005	31	0.002	0.027	0.02	0.024	5.0E-04
**rs10224210**	7	rs6464167	0.42 (A)	0.015	0.021	20	0.003	0.004	0.33	0.010	3.5E-02
**rs4744712**	9	rs1556751	0.59 (A)	0.016	0.0002	65	0.001	0.006	0.19	0.013	2.6E-04
**rs653178**	12	rs12302645	0.94 (A)	−0.018	0.050	10	0.005	−0.014	0.16	−0.017	3.0E-02
**rs626277**	13	rs9318029	0.13(T)	0.016	0.046	7	0.007	0.002	0.42	0.011	8.5E-02
**rs1394125**	15	rs2454472	0.84 (A)	−0.020	0.0009	28	0.002	−0.007	0.25	−0.016	1.4E-03
**rs12460876**	19	rs3795058	0.43 (C)	−0.012	0.004	30	0.002	0.009	0.88	−0.007	6.7E-02
**rs10794720**	10	rs2805575	0.20 (C)	0.013	0.01	45	0.001	−0.016	0.97	0.005	2.3E-01
**rs491567**	15	rs16966247	0.17 (C)	−0.022	0.0002	35	0.001	−0.004	0.31	−0.017	6.0E-04
**rs2453533**	15	rs1153859	0.52 (T)	−0.014	0.001	14	0.004	−0.012	0.04	−0.013	2.2E-04
**rs7422339**	2	rs957749	0.79 (A)	−0.011	0.04	13	0.004	0.007	0.82	−0.006	2.0E-01
**rs2279463**	6	rs3798156	0.12 (T)	−0.027	7.8E-05	22	0.002	−0.032	0.003	−0.028	1.2E-06
**rs6465825**	7	rs6973213	0.20 (A)	−0.019	2.79E-04	25	0.002	−0.008	0.19	−0.017	2.75E-04
**rs10774021**	12	rs485514	0.91 (T)	0.019	0.03	20	0.003	0.013	0.17	0.017	1.6E-02
**rs9895661**	17	rs11650989	0.22 (A)	0.016	0.002	20	0.003	0.013	0.06	0.016	5.8E-04

We also interrogated the CARe CKD GWAS results at the chromosome 22 *MYH9/APOL1* locus (Figure S4); however, none of the G1 haplotype SNPs in *APOL1* was present in our GWAS dataset. The lowest p-value was observed for rs739097 (MAF 0.36, p = 0.00179), which was in weak LD with the previously described rs4821480 in *MYH9* (r2 0.03, D′ 0.38 as determined by Hapmap Release 22 YRI phase 2) [10].

### Genome-Wide Association Analyses for Renal Indices

We performed discovery analyses using GWAS and the IBC chip array; p-value thresholds for discovery were p<5.0*10^−8^ for GWAS and p<2.0*10^−6^ for the IBC array [17]. We observed no genome-wide signals; instead, we carried forward 8 SNPs from GWAS that had a p-value<5*10^−6^ in Stage 1 for replication; results for these SNPs are presented in Table 4.

**Table 4:** Stage 1 and Stage 2 results from loci in African Americans from GWAS (p<5.0*10E-06): SNP association with renal traits.

Trait	SNP ID	Chr	Genes In or Nearby	SNP function	Coded Allele Frequency#	Beta	Stage 1 P-value	Replication beta for lead trait	Stage 2 p-value	Stage 1+Stage 2 beta	Stage 1+Stage 2 p-value
**GWAS**											
UACR	rs4555246	18	DOK6	Intron	0.77 (A)	0.156	8.74E-07	0.078	0.10	0.140	5.33E-07
UACR	rs13213851	6	HACE1	N/A	0.68 (A)	−0.130	1.86E-06	0.102	0.97	−0.084	5.25E-04
UACR	rs2880072	6	FNDC1	N/A	0.66 (A)	−0.128	1.99E-06	−0.097	0.04	−0.122	2.98E-07
MA	rs1009840	6	SGK1	Intron	0.19 (A)	0.363	2.62E-07	0.034	0.36	0.246	1.21E-05
eGFRcrea	rs6581768	12	DYRK2	N/A	0.87 (A)	0.035	3.00E-07	0.002	0.43	0.029	2.62E-06
eGFRcrea	rs7784820	7	GNAT3	N/A	0.70 (A)	0.023	5.30E-07	−0.008	0.87	0.014	2.33E-04
CKD	rs12575381	11	B3GAT1	N/A	0.18 (A)	0.521	9.97E-07	−0.127	0.90	0.179	1.26E-02
CKD	rs6428106	1	RGS1	downstream	0.17(T)	0.549	2.95E-07	0.144	0.07	0.334	3.70E-06
**IBC**											
eGFRcrea	rs7111394	11	KCNQ1	Intron	0.83 (T)	−0.025	4.67E-06	−0.012	0.10	−0.0213	3.61E-06
eGFRcys	rs6865647	5	PDE4D	Intron	0.74 (A)	0.108	1.491E-07	−0.007	0.74	0.0169	6.87E-02
CKD	rs4648006	4	NFKB1	Intron	0.13 (T)	0.433	1.06E-05	−0.140	0.89	0.1886	1.07E-02

**Figure 1:**
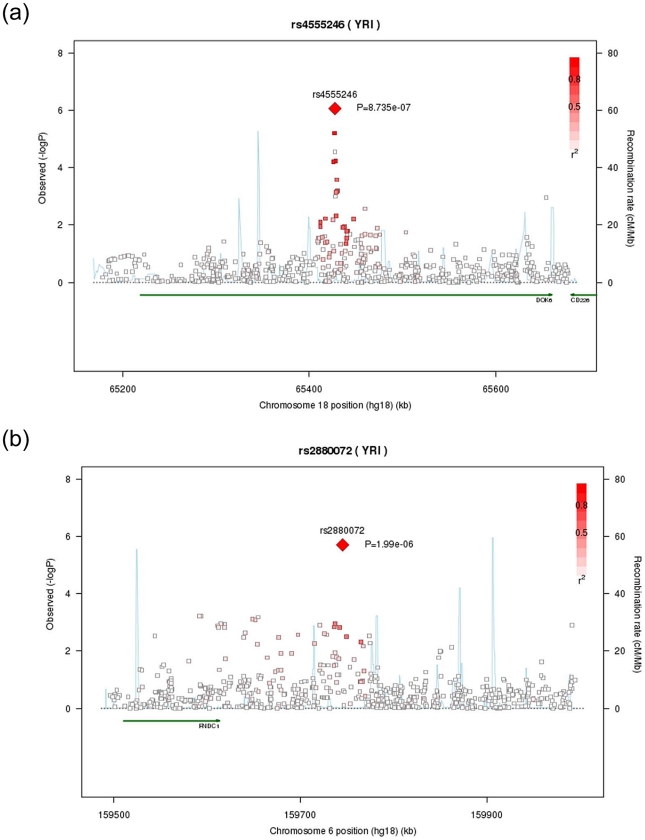
Regional association plots for *DOK6 and FNDC1*. For A) *DOK6* and B) *FNDC1*, Stage 1 only.

Replication was performed in 4,358 participants of African ancestry for eGFR and 2,110 for UACR. Characteristics of the replication samples are shown in Table 1. Results from discovery, replication, and the combined GWAS analyses in African Americans are presented in Table 4 (imputation scores can be found in Table S2). Of the 8 SNPs from GWAS carried forward to replication, the combined Stage 1 + Stage 2 p-value was 5.3*10^−7^ for the association between UACR and rs4555246 in *DOK6* (Figure 1a) and the combined p-value was 2.9*10^−7^ for association between UACR and rs2880072 in *FNDC1* (Figure 1b). Due to modest power of our replication set and the possibility that loci relevant for CKD may be similar across ethnic groups, we also attempted replication of the association between UACR and these two loci in the CKDGen consortium, a large consortium of participants of European ancestry (n = 31,580 with UACR [18]). The beta coefficient for rs4555246 in *DOK6* in the CKDGen data was direction-consistent, although the p-value for this SNP was non-significant (p = 0.44). Recognizing that regions, but not necessarily specific tagging SNPs, may replicate across ethnicities, we interrogated the 250 kb flanking region of rs4555246 (n = 31 independent SNPs). The SNP with the lowest p-value was rs11151530 (MAF 0.17, p = 0.008), which did not meet the Bonferroni-corrected threshold of p = 0.0016 (0.05/31). For *FNDC1*, the beta coefficient for the lead SNP was direction-consistent in CKDGen (beta = −0.0196), although the p-value was not significant (p = 0.053). Interrogating the 250 kb flanking region (n = 7 independent SNPs) revealed that rs7758822 had the lowest p-value at p = 0.002 (MAF 0.12), which met the Bonferroni-corrected threshold of p = 0.007 (0.05/7).

### IBC Chip Analyses for Renal Function Indices

**Figure 2:**
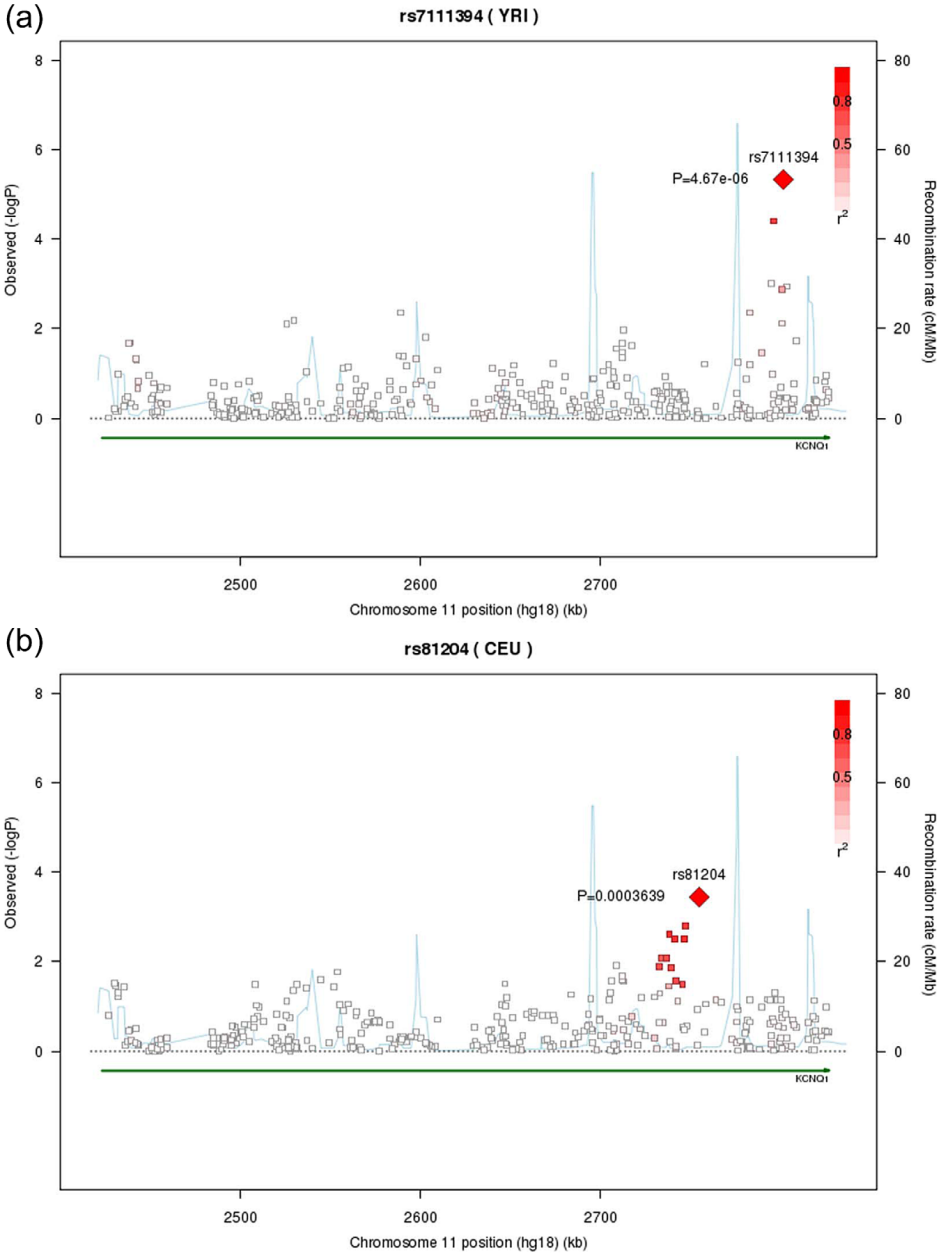
Regional association plots for *KCNQ1*. For A) *KCNQ1* in participants of African ancestry and B) in participants of European ancestry.

In European Americans, we confirmed several known loci for eGFR/CKD [3], [4], [16]; no novel genome-wide significant associations (defined as p<2.0*10^−6^) were identified (Table S3). In African Americans, three loci for eGFR/CKD were brought forward to replication (Table 4). Of these, we observed nominal replication for rs7111394 in the *KCNQ1* gene (Figure 2a, Stage 1 + Stage 2 p-value = 3.6*10^−6^). This SNP was not identified in the European American analysis as it was monomorphic in this population. Thus, we interrogated the 250 kb flanking region around this SNP (93 independent SNPs) in the CARe IBC European ancestry participants. The SNP with the lowest p-value was rs81204 (Figure 2b, MAF 0.16, p-value = 0.00036), which exceeded the corrected regional-specific threshold of 0.000538 (0.05/93).

### Functional Studies of New Loci for Renal Function

To further understand the impact of the three new loci on kidney function and to bolster confidence in the sub-genome-wide statistical associations that we observed, we performed morpholino knockdown of kcnq1, dok6, and fndc1 in zebrafish embryos (see methods).

**Figure 3:**
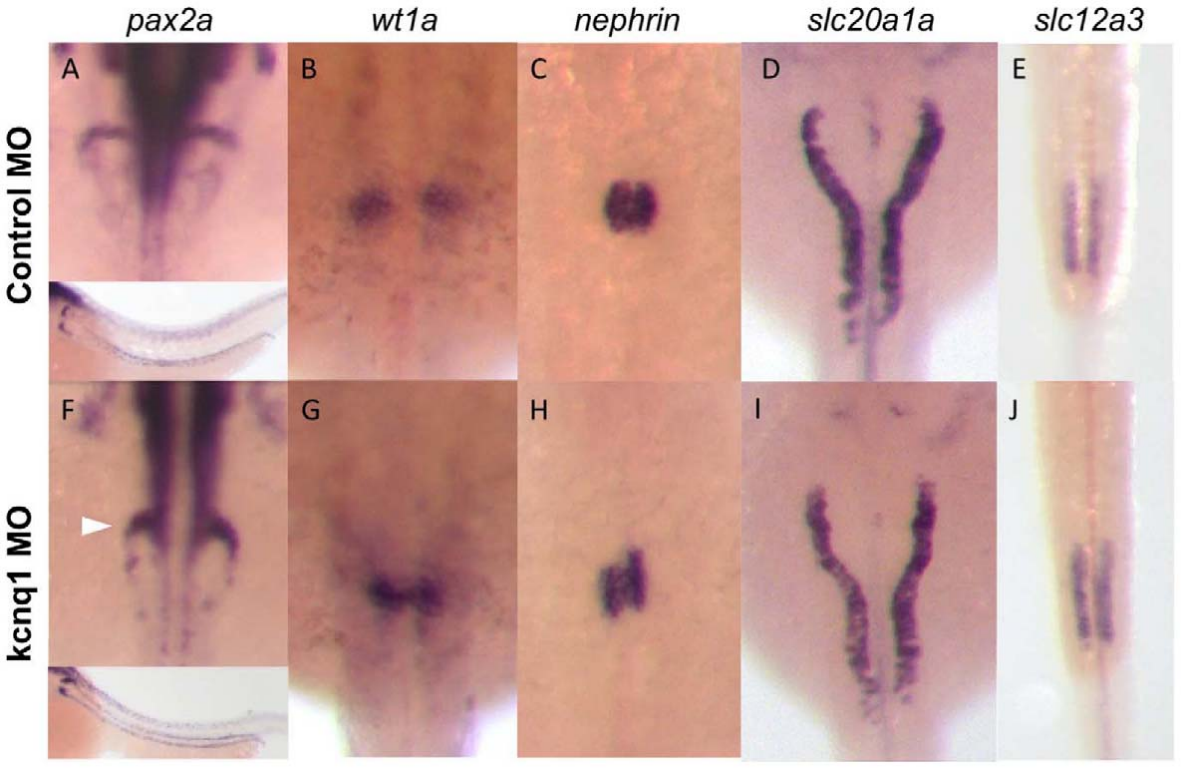
Kcnq1 knockdown in zebrafish embryos causes glomerular abnormalities. (A–E) Zebrafish embryos injected with control morpholino show normal glomerular and tubular morphology, as shown by in situ hybridization for the global kidney marker pax2a (A, inset showing lower-magnification image, with staining in both glomerulus and tubules), the podocyte markers wt1a (B) and nephrin (C), and the proximal and distal tubular markers slc20a1a (D) and slc12a3 (E). (F–J) Injection of the kcnq1 antisense morpholino, targeting the ATG site of the gene, at the one-cell stage results in significant changes in glomerular gene expression (F–H). No changes were observed in the proximal tubule (I) or the distal tubule (J).

*In situ* hybridization for well-established renal markers was used to assess specific anatomic regions of the kidney during development. *Kcnq1* knockdown caused abnormalities in glomerular gene expression in the majority of injected embryos, as shown by the global kidney marker *pax2a* at 48 hours post fertilization (hpf) (see Table 5, Figure 3F). Assessment of the podocyte markers *wt1a* at 24 hpf and *nephrin* at 48 hpf revealed similar, glomerular-specific effects. In contrast, the tubular markers *slc20a1a* and *slc12a3* showed no significant changes. Analysis of glomerular architecture at 120 hpf by electron microscopy did not demonstrate significant differences between control and *kcnq1* morphant embryos (Figure S5), possibly due to diminished morpholino efficacy at this later stage. Knockdown of *dok6* and *fndc1* did not result in generalized edema or significant developmental abnormalities of the kidney (Table 5, Figure S6).

**Table 5:** Number of observed abnormalities/number of embryos examined at 200 uM injection (kcnq1) and 400 uM injection (dok6 and fndc1).

	Global Renal Marker pax2a	Podocyte Marker wt1a	Podocyte Marker nephrin	Proximal Tubular Marker slc20a1a	Distal Tubular Marker slc12a3
Control MO	2/49 (4.1%)	3/63 (4.8%)	2/47 (4.3%)	45/142 (31%)	0/62 (0%)
*kcnq1*					
Splice	45/68 (66%)	30/60 (50%)	35/50 (70%)	54/143 (38%)	0/35 (0%)
ATG	46/68 (68%)	33/78 (42%)	29/46 (63%)	65/157 (41%)	0/41 (0%)
Chi-Sqr p-value*	<0.0001	<0.0001	<0.0001	0.34	1
Chi-Sqr p-value**	<0.0001	<0.0001	<0.0001	0.11	1
Uninjected control	0/38 (0%)	0/31 (0%)	0/60 (0%)	1/16 (6%)	0/18 (0%)
*dok6*					
ATG	5/54 (9%)	3/28 (11%)	1/50 (2%)	5/32 (16%)	2/42 (5%)
Chi-Sqr p-value**	0.14	0.2	0.93	0.65	0.88
*fndc1*					
Splice	4/39 (10%)	1/30 (3%)	0/51 (0%)	8/31 (26%)	0/35 (0%)
Chi-Sqr p-value*	0.13	0.99	1	0.22	1

*Control or Uninjected versus splice MO.

**Control or Uninjected versus ATG MO.

**Figure 4:**
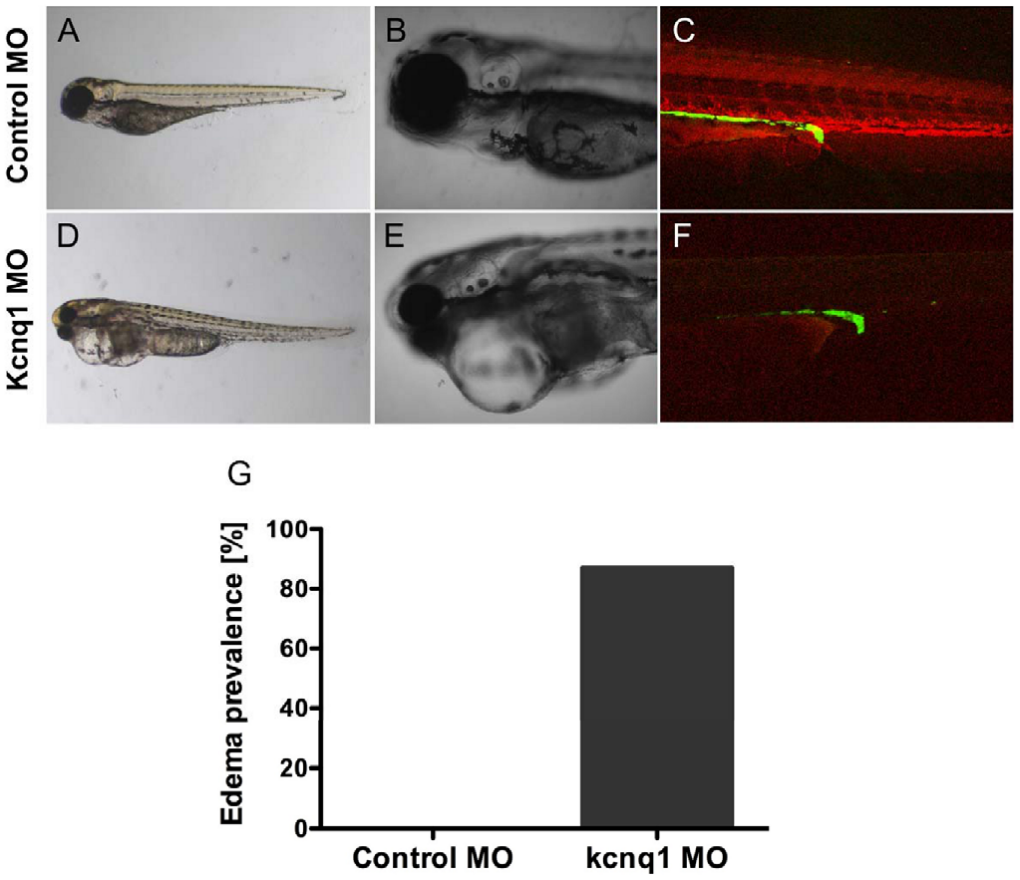
*Tg(cdh17:GFP)* embryos were injected with 200 uM control or kcnq1 morpholino (MO) at the 1-cell stage. Embryos were subsequently injected in the sinus venosus with 10,000 MW rhodamine dextran at 48 hpf. Dextran clearance was monitored over the next 48 hours by confocal microscopy. Compared to control MO-injected embryos (A–C), kcnq1 MO-injected embryos exhibit edema (D, E) and increased dextran clearance (F) 48 hours post-injection (hpi), suggestive of a filtration defect. At 5 dpf, 87% of kcnq1 morphant embryos (n = 38) develop edema (G).

To determine whether differences in gene expression resulted in altered kidney function, we evaluated glomerular filtration in *kcnq1* morphant embryos by assessing the kidney's capacity to retain fluorescent dextran. Fluorescently labeled high-molecular weight dextran has been used in zebrafish to directly visualize functional glomerular integrity [19]. Control or *kcnq1* morphant embryos were equally loaded with rhodamine-labeled 10,000 MW dextran by injection into the cardiac sinus venosus at 48 hpf (Figure S7A, S7D). Dextran clearance was assessed by overall fluorescence in the embryo at 72 and 96 hpf (Figure S7B, S7C, S7E, S7F) and presence of red fluorescence in the green fluorescent tubules of *cdh17:GFP* reporter embryos. *Kcnq1* morphant embryos exhibited decreased fluorescence by 96 hpf, indicative of increased dextran clearance compared to control embryos (Figure 4C, 4F). Time course analysis confirmed equal loading and progressive loss of fluorescence over 48 hours in *kcnq1* morphants. These results suggest that loss of kcnq1 causes decreased glomerular retention of macromolecules. The majority of embryos with increased loss of dextran fluorescence also exhibited generalized edema (Figure 4A, 4B, 4D, 4E, 4G), which has been previously linked to kidney dysfunction in zebrafish [20], [21].

### Cross-Trait Associations

rs4555246 in *DOK6* was associated with albuminuria (OR = 1.20, p = 0.001) but not with eGFR (p = 0.23) or CKD (p = 0.92; Table S4). Similarly, rs2880072 in *FNDC1* was associated with albuminuria (OR = 0.83, p = 4.9*10^−5^) but not with eGFR or CKD. Although rs7111394 in *KCNQ1* was associated with eGFR, it was not significantly associated with CKD (direction consistent OR 1.07, p = 0.36), UACR, or albuminuria. These findings underscore the specificity of the genetic underpinnings of eGFR as compared to albuminuria.

### Stratification by Hypertension and Diabetes Status

All replicating loci were stratified by hypertension and diabetes status (Table S5). We observed nominal significance for nearly all loci in the non-diabetes and non-hypertension strata, and many loci retained statistical significance in the diseased strata despite smaller sample sizes.

## Discussion

In more than 8,000 African Americans, for 24 known renal susceptibility loci identified in European ancestry consortia for eGFR and CKD, we have identified the most significant SNP in African Americans, of which several showed evidence of confirmation or replication. In addition, we performed discovery analyses using GWAS and a candidate-gene based array, and uncovered 3 suggestive loci, including *KCNQ1* in association with eGFR and *DOK6* and *FNDC1* in association with UACR. Finally, we show that loss of function of *kcnq1* leads to abnormalities in glomerular gene expression and function during zebrafish development.

Despite having one of the largest GWAS datasets in African Americans, both our discovery and replication samples were of relatively modest size. Thus, we have focused primarily on interrogating regions previously identified in well-powered European ancestry meta-analyses for renal function in order to reduce our genome-wide penalty for multiple testing. While we have uncovered 3 suggestive loci, the strength of the statistical significance is only suggestive. Because of this, we have corroborated our findings with functional data from zebrafish, providing compelling evidence for the role of *KCNQ1* in renal abnormalities.

Several prior genome-wide studies using GWAS or admixture approaches have identified loci for renal function in participants of European [3], [4], [16] and African ancestry [10], [11]. These studies have identified loci for eGFR, CKD, and non-diabetic ESRD; however, few novel discoveries and relatively limited replication have been made for renal function indices in African Americans. African ancestry populations have smaller LD blocks and hence more genetic diversity, thus offering the potential opportunity to fine map genomic regions and to identify novel genomic regions for indices of renal function.

The genes located closest to our three suggestive loci are *KCNQ1* in association with eGFR, as well as *FNDC1* and *DOK6* in association with UACR. *KCNQ1* on chromosome 11 encodes for the potassium voltage-gated channel, KQT-like subfamily, member 1 (KvLQT1). The lead SNP identified among the African ancestry participants is located in an intron of *KCNQ1* and is flanked by two recombination hotspots. The kcnq1 protein is abundantly expressed in the brush border membrane of renal proximal tubule cells, where it interacts with other K^+^ channels (KCNE) to mediate net K^+^ secretion. Loss of Kcnq1 in the mouse leads to impaired Na+ absorption with increased glucose load, however, developmental abnormalities have not been reported [22]. Variants in *KCNQ1* have been associated with type 2 diabetes [23] and beta cell function [24], due to its role as a potassium channel in the pancreatic beta cells. A recent study of individuals with diabetes from Japan identified variants in the *KCNQ1* gene in association with diabetic nephropathy [25]. Our lead SNP is not in LD with this signal in either European or African ancestry individuals. Importantly, we observe a strong and significant association with *KCNQ1* among participants without diabetes, and our functional work suggests an independent association of *kcnq1* in renal development. Thus, it is unlikely that our observed associations are solely due to the association of *KCNQ1* with diabetes.

The *DOK6* gene on chromosome 18 encodes a member of a family of intracellular adaptor proteins that have a role in the assembly of multimolecular signaling complexes. It is expressed in the human kidney as well as the ureteric buds of the murine developing kidney [26]. Although a direct role in renal disease has not been described so far, DOK6 interacts with Ret [26], the ablation of which resulted in kidney agenesis in model systems [27]. The lead SNP we identified is located in an intron of a very circumscribed region of the gene. *DOK6* has been recently found in a GWAS for osteoporosis [28], although our lead SNP is not in LD with this variant.

The fibronectin type III domain containing 1 (*FNDC1*) gene on chromosome 6 is also expressed in the kidney; the lead SNP is located nearly 100 kb downstream of the gene. Little is known about the function of this gene to date; previous studies found that FNDC1 may mediate G protein signaling and have a role in hypoxia-induced apocytosis of cultured ventricular cardiomyocytes [29], [30].

Zebrafish have been extensively used to study principal pathways of kidney development and function [31], [32]. More recently, adult models of kidney injury have been developed [33]. For example, targeted knockdown of a prolyl 4-hydroxylase resulted in kidney dysfunction with edema and changes in podocytes and Bowman's capsule [34]. In addition, a zebrafish model of human nephrotic syndrome was generated by *plce1* knockdown after positional cloning of this gene in affected siblings, similarly resulting in cardiac edema and functional abnormalities [35]. Here, we use morpholino knockdown to demonstrate that loss of *kcnq1* leads to changes in global morphology and gene expression abnormalities during zebrafish kidney development. Furthermore, in vivo fluorescence-based functional analysis of zebrafish glomerular filtration capacity demonstrated decreased retention of macromolecules, as previously demonstrated for other genes affecting glomerular integrity [19], [20]. Interestingly, *KCNQ1* was identified in association with eGFR, and knockdown of *kcnq1* in zebrafish predominantly causes glomerular gene expression and filtration defects. These results suggest that genes associated with polygenic chronic conditions can produce developmental phenotypes when knocked down *in vivo.*

Our analytic approach was complemented by a large-scale candidate gene analysis using the IBC SNP Chip array [15] and replication of our findings in an additional 4358 African American individuals. By using participants from predominantly population-based cohorts, we were able to study disease initiation. An important focus of our multi-ethnic samples allowed us to explore allelic heterogeneity, and support the notion that genomic risk regions are observed across ethnicities. Our replication was derived from *in silico* samples, which allowed for the adjustment for principle components where necessary. Finally, we were able to corroborate our results in the zebrafish, a model organism for studying vertebrate kidney development.

While the advantage of the zebrafish model is to rapidly assess gene function during development, the role of genes in aging and chronic disease cannot be modeled by transient morpholino knockdown. eGFR was estimated using the MDRD study equation, as obtaining gold-standard measures of GFR in large population-based samples is not feasible. Serum creatinine and albuminuria were measured at a single-point in time, which may misclassify certain individuals and bias our results toward the null. Even with imputation, the coverage of the genome in African Americans is not comprehensive, thus limiting our statistical power. With respect to the chromosome 22 *MYH9/APOL1* locus, neither the E1 haplotype in *MYH9* nor the G1 haplotype in *APOL1* were in our GWAS dataset, limiting our ability to examine this region in association with more modest CKD phenotypes. Finally, our power was low for CKD to detect SNPs with odds ratios of 1.2, the largest odds ratio detected in other GWAS for CKD [4].

We identified several SNPs in association with eGFR in African Ancestry individuals, as well as 3 suggestive loci for UACR and eGFR. Functional genomic studies support a role for *kcnq1* in glomerular development and function in zebrafish.

## Methods

### Renal Function Indices

Serum creatinine was measured as described in Text S1. Serum creatinine was calibrated to NHANES in all studies (including replication cohorts) to account for between-laboratory variation as previously described [3], [36], [37]. Glomerular filtration rate was calculated based on serum creatinine (eGFR) with the Modification of Diet in Renal Disease (MDRD) equation [38]. We defined chronic kidney disease (CKD) as eGFR <60 ml/min/1.73 m^2^ in accordance with the National Kidney Foundation guidelines; CKD was based on a single serum creatinine measurement as described in Text S1. Urinary albumin to creatinine ratio (UACR, mg/g) was computed as described in Text S1; microalbuminuria was defined as UACR >17 mg/g [men] and >25 mg/g [women].

### Covariate Definitions

We defined diabetes as fasting glucose ≥126 mg/dl, self-report, or pharmacologic treatment. Similarly, hypertension was defined as systolic blood pressure ≥140 mm Hg, diastolic blood pressure ≥90 mm Hg, or pharmacologic treatment.

### Genotyping Platforms for Genome-Wide Genotype and Imputation

For the present study, the CARe consortium genotyped the IBC SNP chip [15] in 23767 European Americans and 8110 African Americans, as well as the Affymetrix 6.0 chip in 7382 African Americans. The IBC array contains nearly 50,000 SNPs across 2,000 loci. SNPs were selected using a tagging approach among populations represented in HapMap and the SeattleSNPs project. The array was designed to focus on candidate loci related to cardiovascular disease and its risk factors. More details can be found in the design paper [15]. Table S1 details the genotyping that was conducted. For the CARe study cohorts, quality control and imputation were conducted centrally using MACH 1.0.16 (http://www.sph.umich.edu/csg/abecasis/MaCH/). Imputation results were filtered using thresholds RSQ_HAT value of 0.3 and minor allele frequency 0.01. Fractional counts between 0 and 2 were coded for the imputed genotypes in order to estimate the number of copies of a pre-specified allele. For European samples, the CEU population from HapMap 2 (2.54 million SNPs) was used as the reference panel.

For African American samples, a 1∶1 combined HapMap 2 CEU+YRI reference panel was used. This panel includes SNPs that were present in both populations, as well as SNPs segregating in one panel and monomorphic and nonmissing in the other (2.74 million altogether). Since the African American samples were genotyped for both the Affymetrix 6.0 and IBC arrays, we were able to analyze imputation performance at non-genotyped SNPs. The use of the CEU+YRI panel resulted in an allelic concordance rate of ∼95.6%, calculated as 1 – 1/2*|imputed_dosage – chip_dosage| for imputation on the Affymetrix chip. This is similar to rates obtained from African ancestry participants imputed using HapMap 2 YRI individuals [39].

### Statistical Methods for Discovery Stage

Trait creation details are described above (Renal Function Indices). Performed centrally but within each individual study, genome-wide association analyses and IBC chip analyses of natural log-transformed eGFR, UACR, CKD, and MA were conducted using linear and logistic regression with an additive genetic model. We adjusted for age, sex and study site (when applicable) and the first 10 principal components; relatedness was accounted for when necessary using linear mixed effect (LME) models for eGFR and UACR and logistic regression via generalized estimating equations (GEE) for CKD and MA. Additional details regarding the discovery cohorts are in Text S1.

### Principal Components Analysis

Principal components were generated using EIGENSTRAT [40] within each study using the CARe African ancestry Affy6.0 genotype data. Two reference populations were included in the principal component analysis of African Americans: 1,178 European Americans from a multiple sclerosis GWA study (from Dr. Phil de Jager and colleagues), and 756 Nigerians from the Yoruba region from a hypertension GWA (provided by Dr. Richard Cooper and colleagues). Importantly, these two underwent extensively quality control procedures to remove population outliers using PCA. Ten principal components were generated for each study and used to adjust for population substructure.

### Meta-Analysis

We performed fixed-effect meta-analyses of the IBC chip and genome-wide association data using the inverse-variance weighted approach in METAL (http://www.sph.umich.edu/csg/abecasis/Metal/index.html). Genomic control correction was applied after calculating the inflation factor lambda (λ) within each individual study and after the genome-wide association meta-analysis was performed.

The standard threshold of p<5×10^−8^ for genome-wide significance in the genome-wide association and p<2.0×10^−6^ in the IBC chip analyses was used. The rationale for the p-value threshold used for the IBC chip is based on an empiric test of the number of independent loci (∼25,000) that appear on the IBC array [17]. We selected independent SNPs (pairwise r^2^<0.2) at each locus for replication.

The R software (v2.9.0) was used for data management, statistical analyses and graphing.

### Interrogation of CKDGen Loci in CARe

We developed a set of criteria to validate the lead SNPs and interrogate regions around each of the loci that were previously reported among European ancestry (EA) participants [3], [4] in our African American (AA) CARe samples. For each lead SNP in EA, we looked-up the respective association result with eGFR in AA. To accommodate the difference of LD structure and possible allelic heterogeneity across different ethnicities, we then interrogated the 250 kb flanking region around each lead SNP to determine whether there exist other SNPs with stronger associations with the outcome. We used the following criteria to identify the top AA SNP: 1) the SNP with the smallest association p-value within the region; 2) MAF >0.03; 3) location of the AA lead SNP within the same recombination block of the lead EA SNP, where the recombination block was defined as a 20% recombination rate. The statistical significance of each identified SNP was evaluated using a region-specific Bonferroni correction. We determined the number of independent SNPs based on the variance inflation factor (VIF), which was calculated recursively within a sliding window with size 50 SNPs and pairwise r^2^ value of 0.2 using PLINK.

Finally, each identified top SNP in AA was sent for replication in additional independent AA samples.

### Interrogation of CARe Loci in CKDGen

Similar to the interrogation in AA for the EA lead loci, we also interrogated the newly identified loci from the CARe GWAS and the IBC chip in EA participants of the CKDGen consortium.

### Stage 2 Replication Analysis

Replication analyses were performed using imputed *in silico* genome-wide association data; replication studies conducted the same association analyses as the Stage 1 phase. Details regarding the replication cohorts can be found in Text S1.

Replication was performed as follows: meta-analysis was conducted in the Stage 2 studies only, and then in the Stage 1 + Stage 2 studies combined. Replication was defined as a direction-consistent Stage 2 beta coefficient; replication p-values are thus represented as one-sided tests. SNPs were declared to replicate when the p-value in the Stage 1 + Stage 2 studies combined was smaller than the p-value in the Stage 1 alone.

### Cross-Trait Analyses

The correlation between ln(UACR) and ln(eGFR) can range from non-significant to as high as 0.237 (p<0.001) in our study; therefore, we examined the cross-trait associations across albuminuria and eGFR phenotypes.

### Functional Studies in Zebrafish

Zebrafish were maintained in accordance with established procedures and IACUC approval. At the one-cell stage, zebrafish embryos were injected with varying doses of morpholino antisense oligonucleotides (MO, GeneTools, Philomath OR). MO sequences are shown in Table S6. For *in vivo* observations, edema development was documented at 5-days post-fertilization.

*In situ* hybridization was performed according to established procedures (http://zfin.org/ZFIN/Methods/ThisseProtocol.html).To visualize different regions of the kidney, we used *pax2a* (global kidney marker) [41], *wt1a* (podocyte marker) [41], *nephrin* (podocyte marker) [31], *slc20a1a* (proximal tubule) [42], and *slc12a3* (distal tubule marker) [42]. The morphology of the expression pattern was independently scored by two investigators. Electron microscopy was performed as previously described [41].

Dextran clearance was assessed as described previously [20], 48 hours after morpholino injection, embryos were manually dechorionated, anesthetized in a 1∶20 dilution of 4 mg/ml Tricaine in egg water and positioned on their back in a 1% agarose injection mold. An equal volume of tetramethylrhodamine dextran (10,000 MW; Invitrogen) was injected into the cardiac sinus venosus of each embryo, after which embryos were returned to fresh egg water. Embryos were imaged by fluorescence microscopy 6 hours post-injection (54 hpf) to demonstrate equal loading, then subsequently imaged at 72 and 96 hpf to evaluate dextran clearance. Confocal images were obtained from agarose embedded embryos using a Zeiss LSM500 microscope.

## Supporting Information

Figure S1 Quantile-quantile plots for genome-wide association for A) eGFR; B) CKD; c) UACR; 4) MA.
(TIF)

Figure S2 Manhattan plots for genome-wide association for A) eGFR; B) CKD; c) UACR; 4) MA.
(TIF)

Figure S3 Regional association plots for all confirmed or replicating loci from the CKDGen loci interrogation; the blue notation represents the best SNP in whites with the p-value in African Americans, whereas red represents the lead SNP in African ancestry participants; the linkage disequilibrium shown uses YRI information from Hapmap2.
(PDF)

Figure S4 Regional association plot for the *MYH9-APOL1* region in African ancestry participants.
(TIF)

Figure S5 Analysis of glomerular architecture after kcnq1 knockdown by electron microscopy does not reveal significant changes. (a–c) Glomerular architecture at 120 hpf after injection of control morpholino visualized by electron microscopy at 8000-, 15,000, and 50,000-fold magnification reveals endothelial capillaries (C) with basement membrane (BM), podocytes (P) and foot processes (FP) similar to mammalian glomerular ultrastructure. (d–f) Transient knockdown of kcnq1 does not significantly change glomerular anatomy.
(TIF)

Figure S6 Dok6 and fndc1 knockdown in zebrafish embryos does not affect kidney development. (a–e) Uninjected control zebrafish embryos show normal glomerular and tubular morphology, as shown by in situ hybridization for the global kidney marker pax2a (a, inset showing lower-magnification image, with staining in both glomerulus and tubules), the podocyte markers wt1a (b) and nephrin (c), and the proximal and distal tubular markers slc20a1a (d) and slc12a3 (e). (f–o) Injection of dok6 (f–j) or fndc1 (k–o) morpholinos at the one-cell stage results in no significant changes in glomerular or tubular gene expression.
(TIF)

Figure S7 kcnq1 knockdown enhances loss of fluorescent dextran. Control and kcnq1 MO injected embryos were loaded with rhodamine-labeled dextran at 48 hpf. >80 embryos were analyzed for each group in 3 separate experiments (a,d) Fluorescence microscopy at 6 hpi reveals equal fluorescence loading between embryos. (b,e) At 72 hpf (24 hpi), fluorescence intensity in kcnq1 morphants is diminished. (c,f) At 96 hpf (48 hpi) control embryos retain their fluorescence, but kcnq1 morphants have significantly diminished fluorescence.
(TIF)

Table S1 Genotyping and imputation platforms.
(DOC)

Table S2 Genome-wide significant loci: SNP imputation quality* in Discovery and Replication cohorts.
(DOC)

Table S3 Known loci for eGFRcrea and eGFRcys among participants of European Ancestry present on the IBC chip.
(DOC)

Table S4 Cross-trait associations for novel loci from Stage 1+Stage 2 in participants of African ancestry.
(DOC)

Table S5 Diabetes and Hypertension stratified analyses, Stage 1 data.
(DOC)

Table S6 Morpholino sequences.
(DOC)

Text S1 Study-specific methods.
(DOC)
