# Supplementary figures and images for: Genetic Association for Renal Traits among Participants of African Ancestry Reveals New Loci for Renal Function
Source: PLoS Genet. 2011 Sep 8;7(9):e1002264. doi: 10.1371/journal.pgen.1002264 (PMC3169523; doi:10.1371/journal.pgen.1002264)

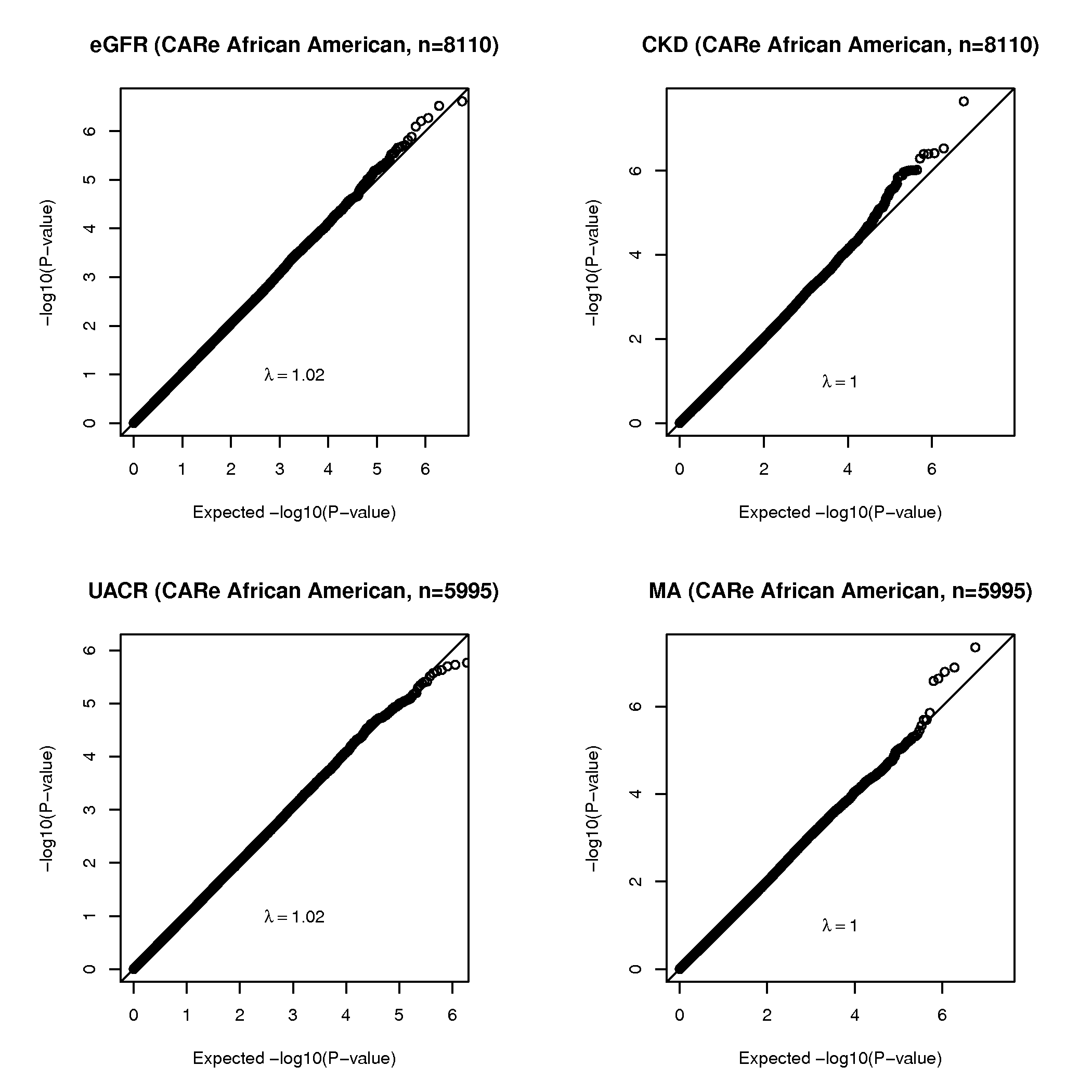

Supplement: Figure S1 — Quantile-quantile plots for genome-wide association for A) eGFR; B) CKD; c) UACR; 4) MA. (TIF) [file pgen.1002264.s001.tif]

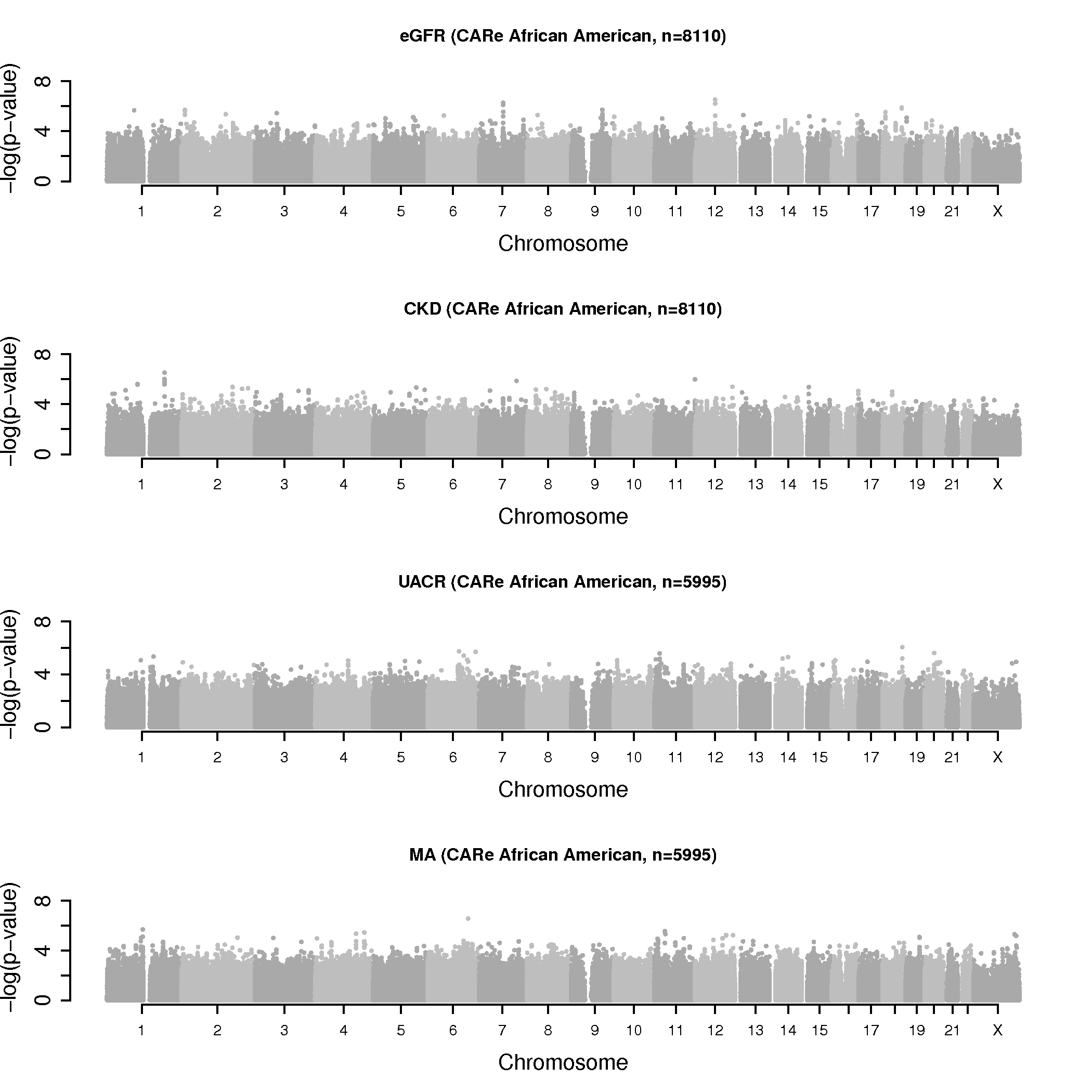

Supplement: Figure S2 — Manhattan plots for genome-wide association for A) eGFR; B) CKD; c) UACR; 4) MA. (TIF) [file pgen.1002264.s002.tif]

rs267734 ( YRI )

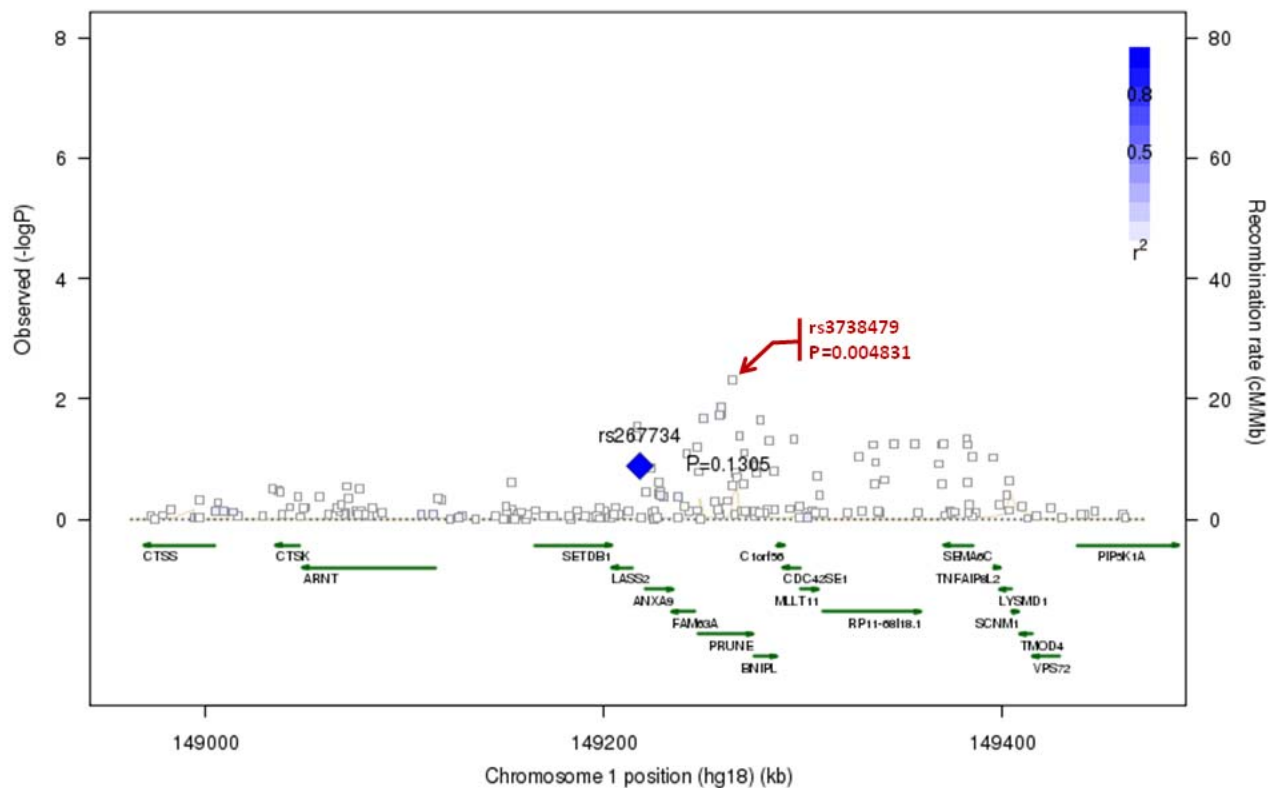

rs1260326 ( YRI )

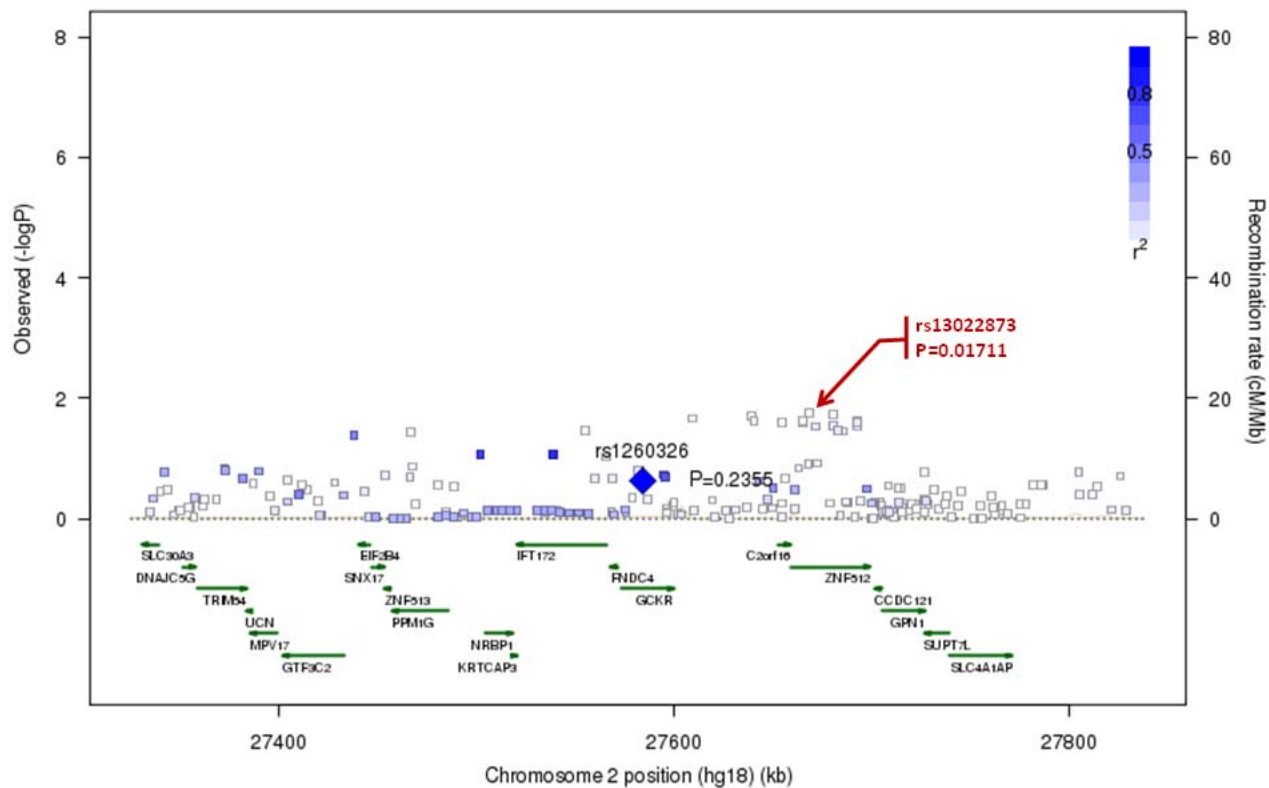

rs347685 ( YRI )

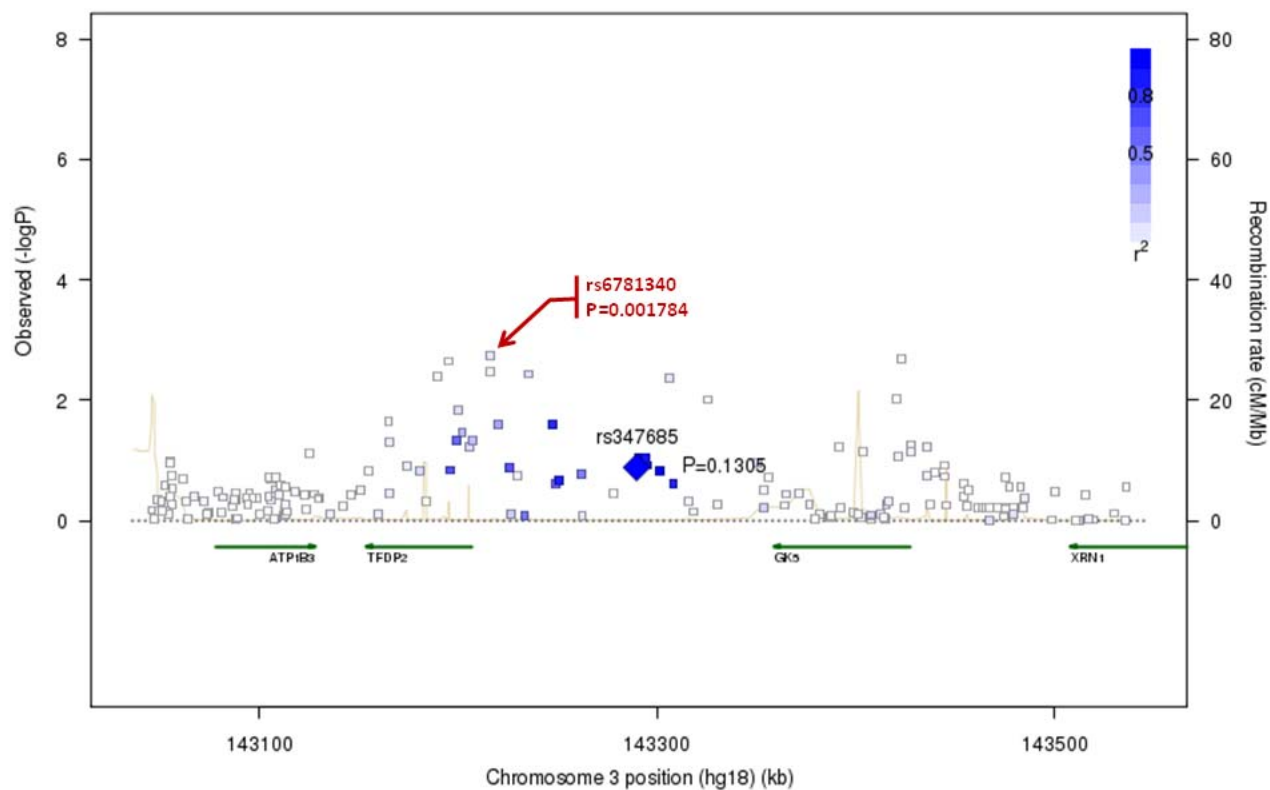

rs11959928 ( YRI )

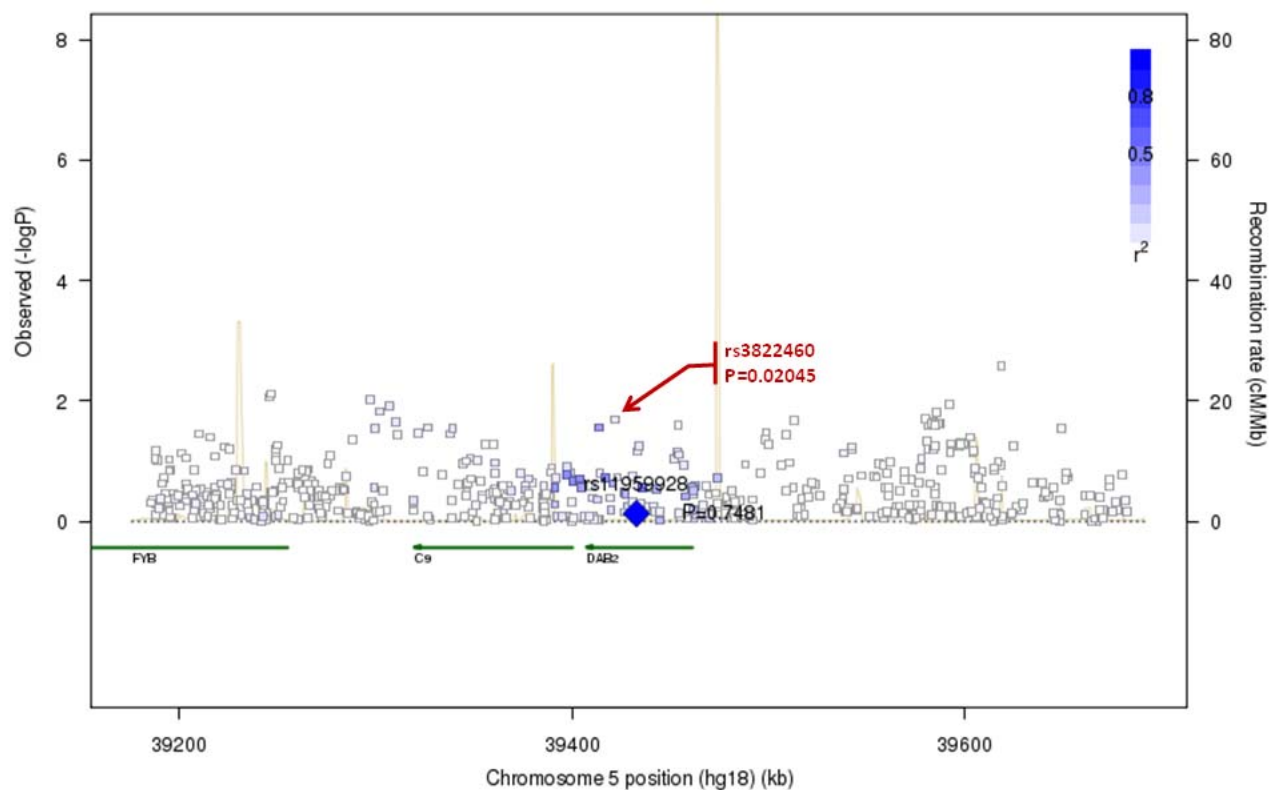

rs881858 ( YRI )

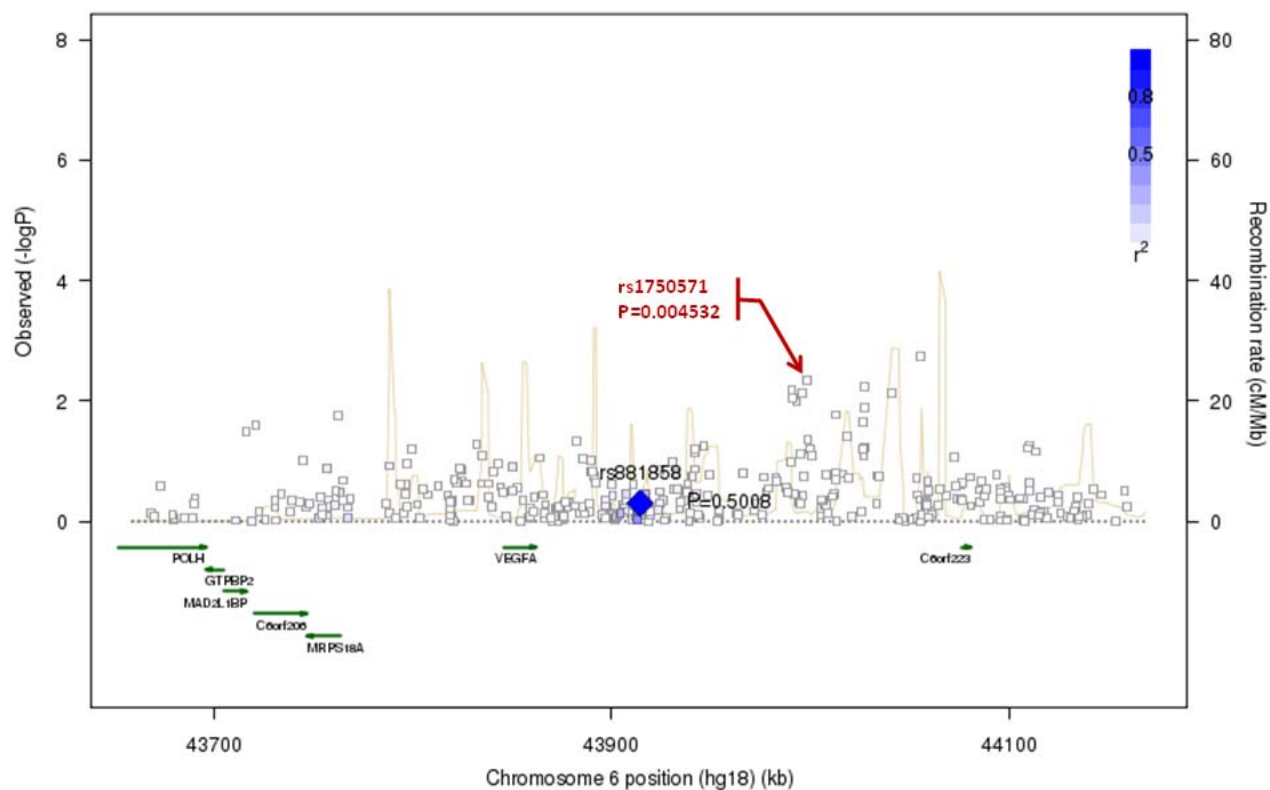

rs2279463 ( YRI )

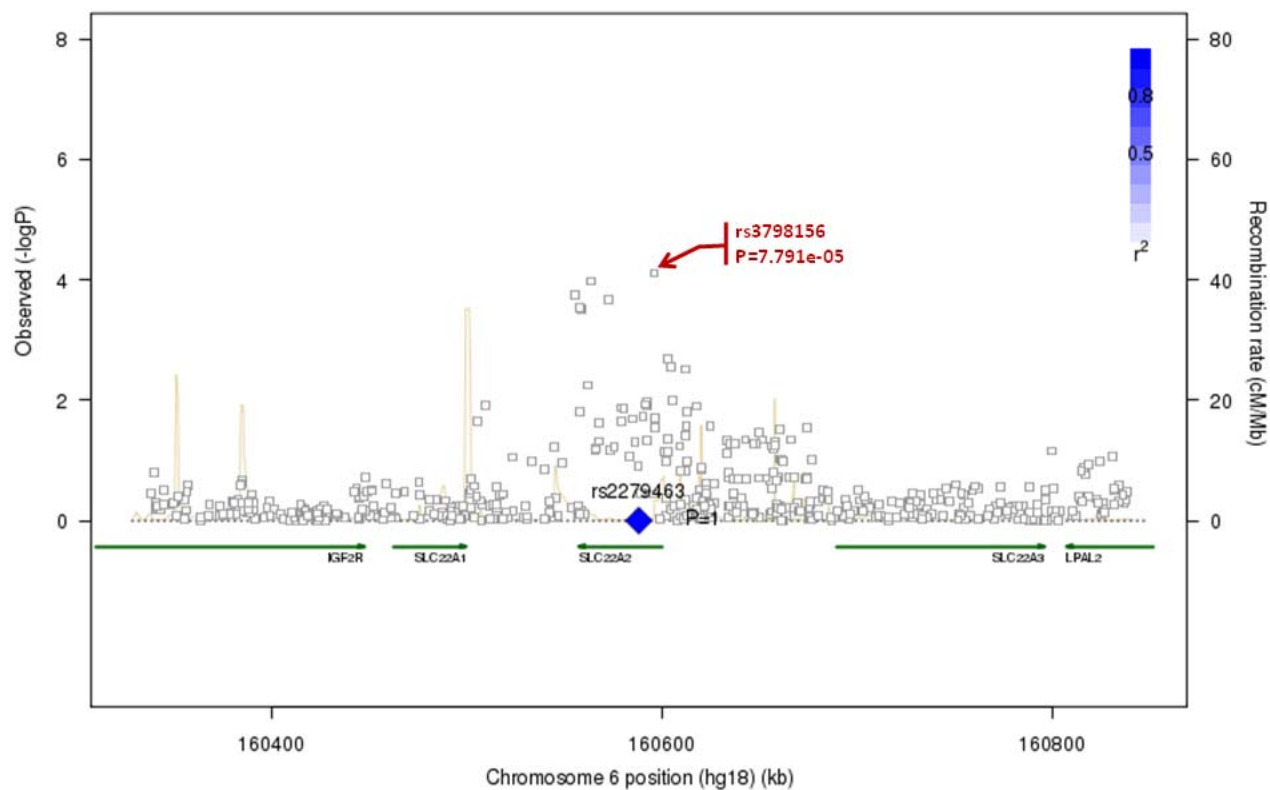

rs6465825 ( YRI )

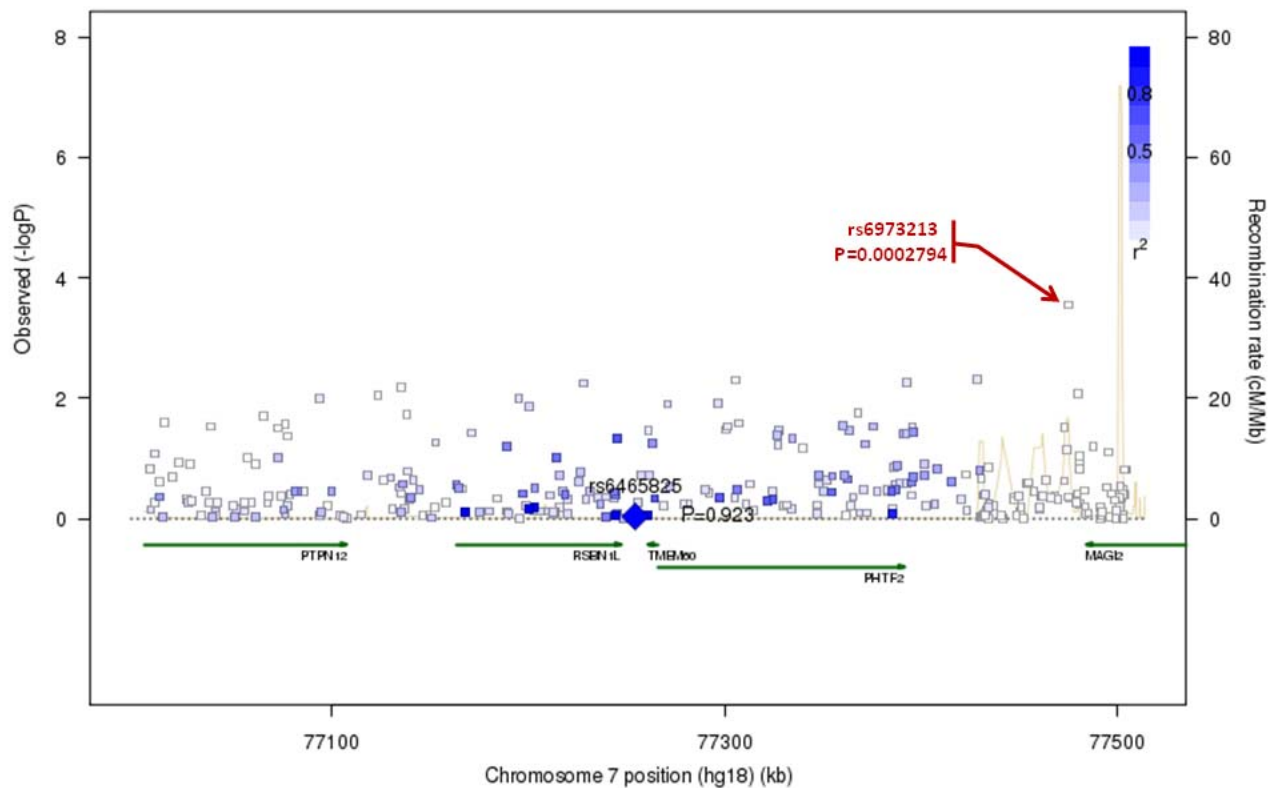

rs4744712 ( YRI )

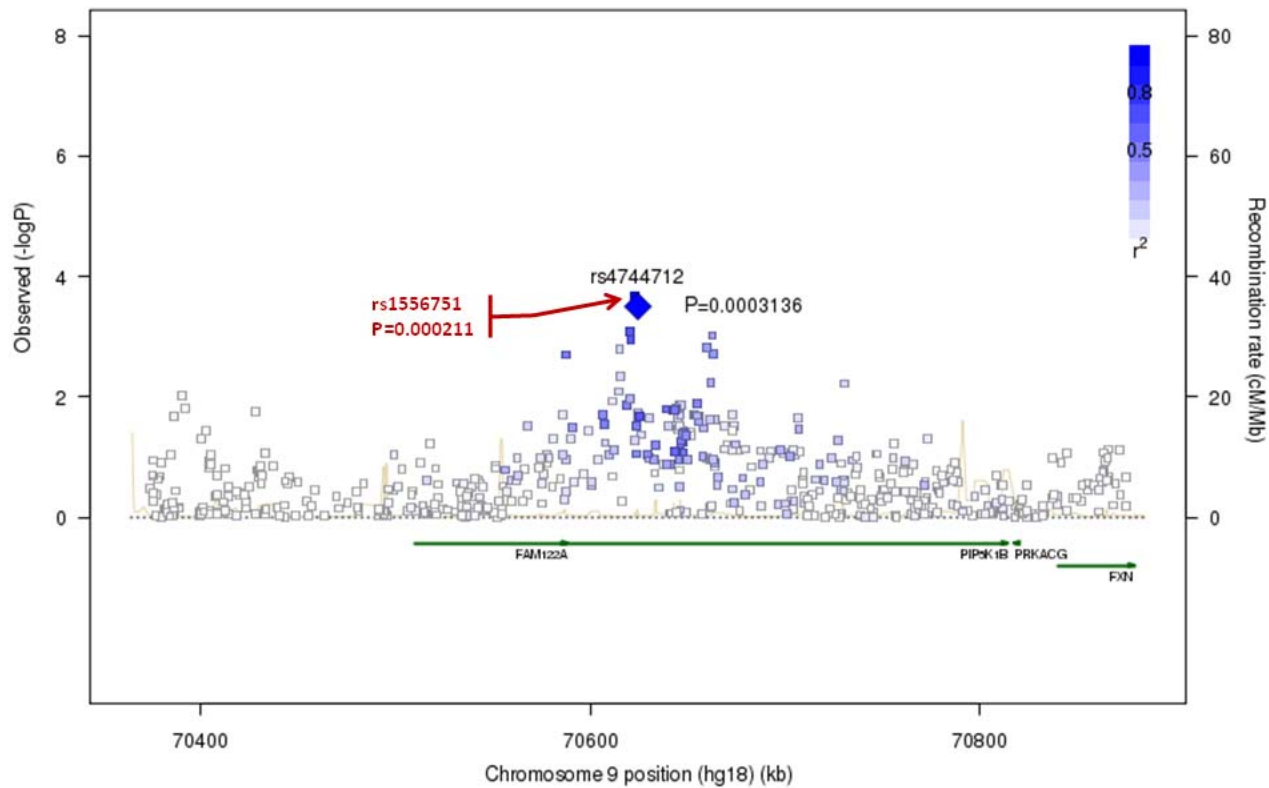

rs10774021 ( YRI )

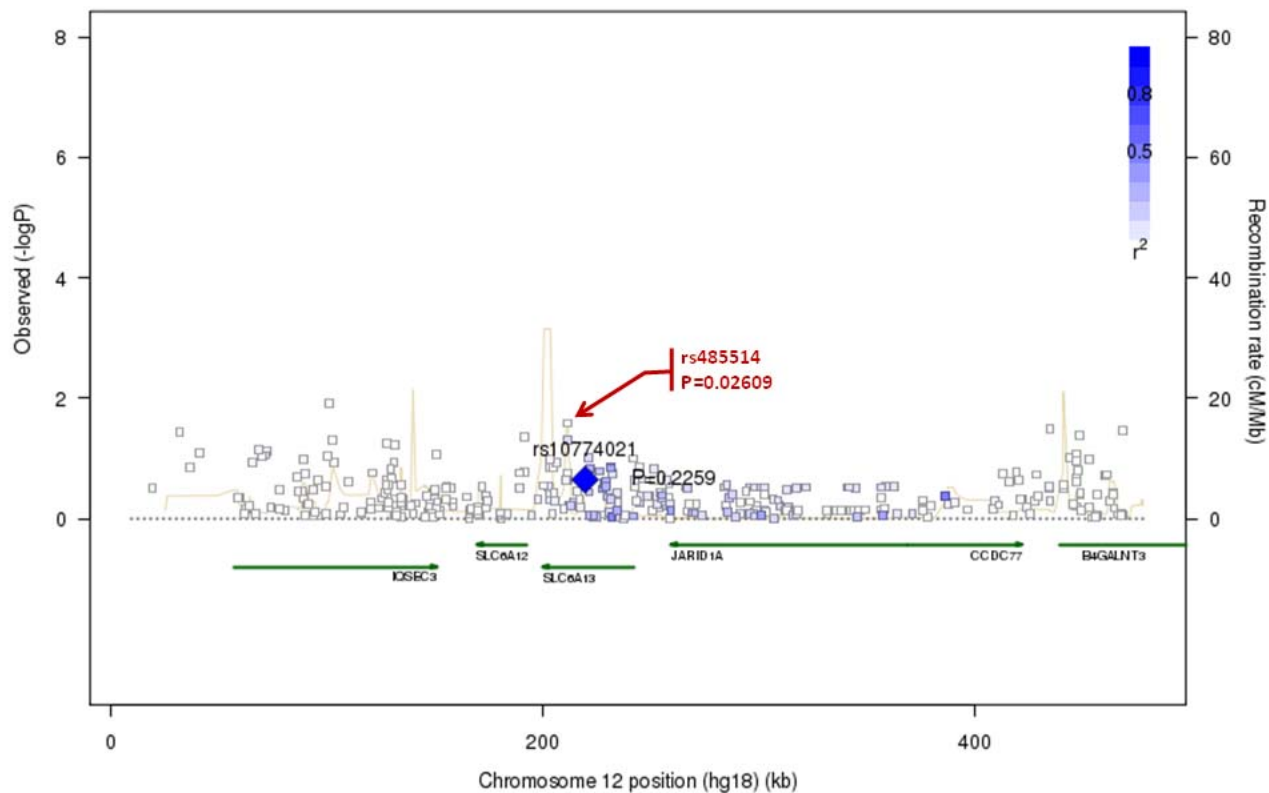

rs653178 ( YRI )

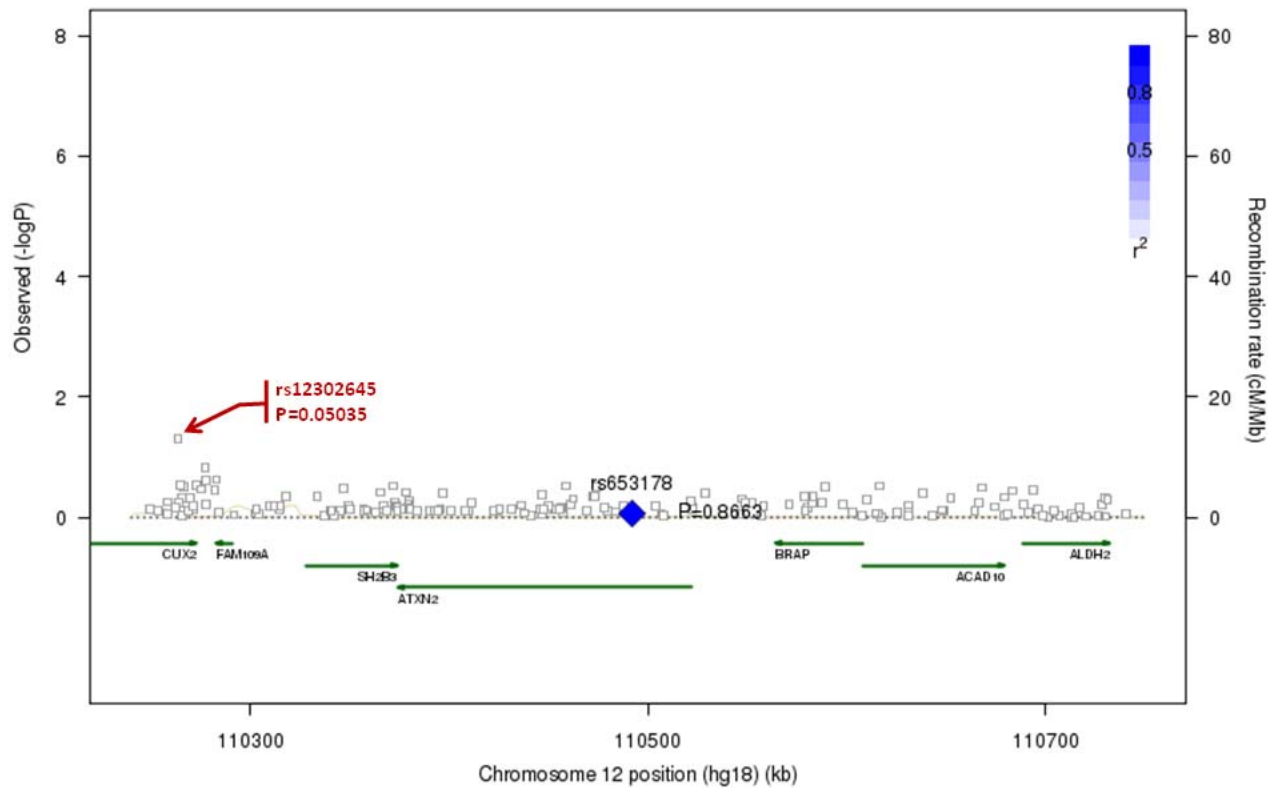

rs2453533 ( YRI )

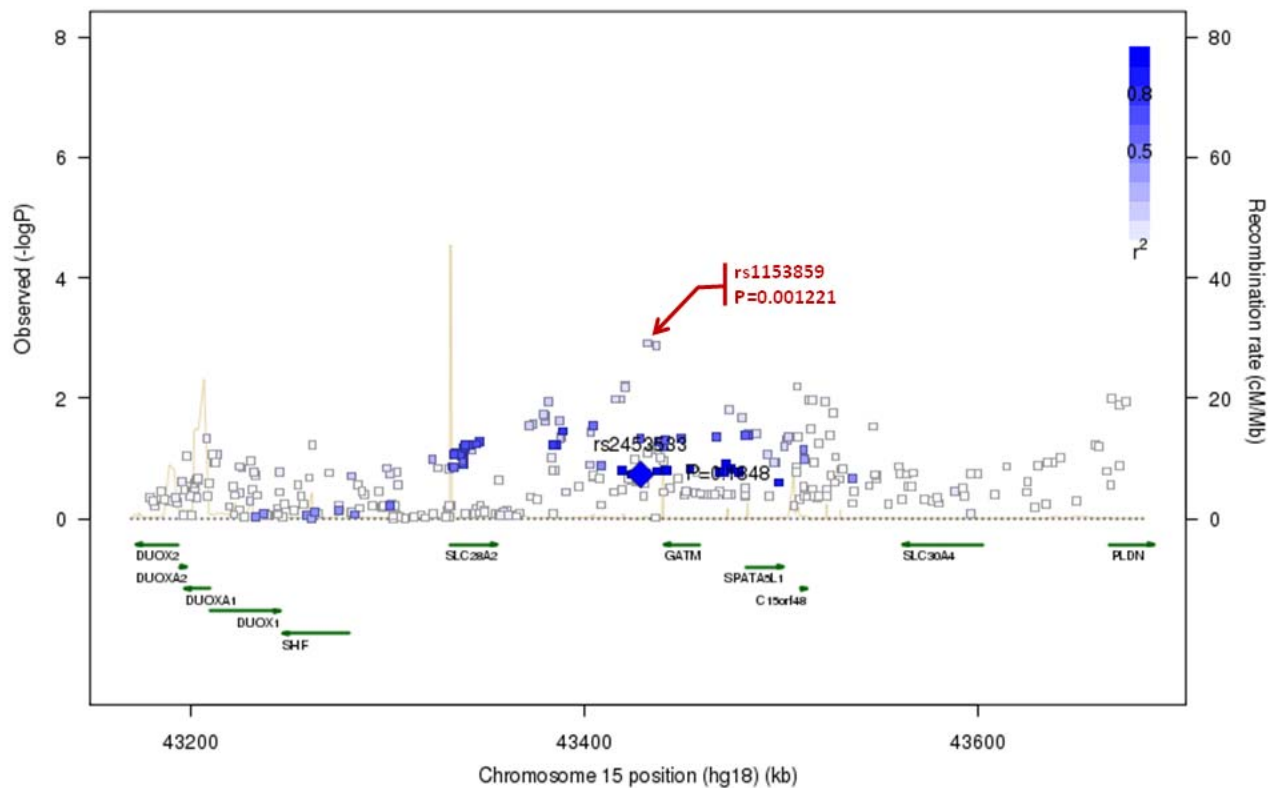

rs491567 ( YRI )

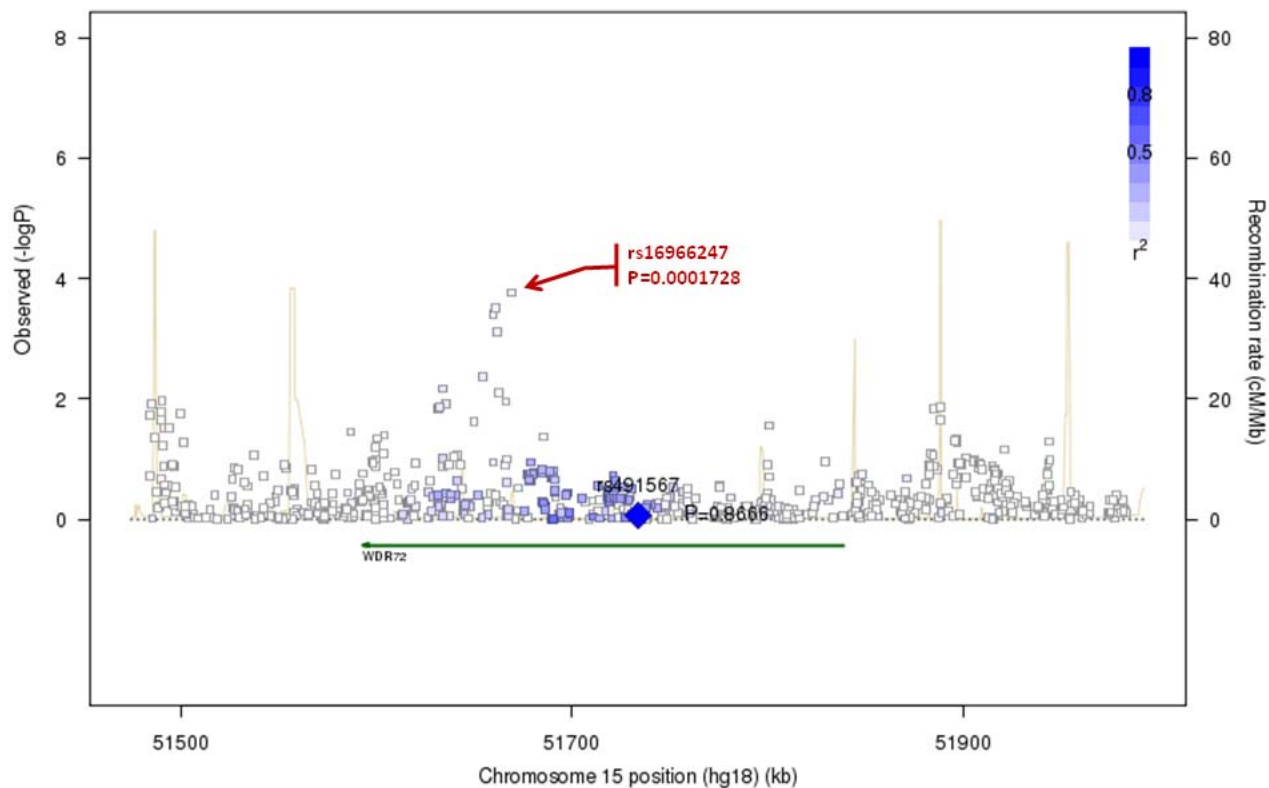

rs1394125 ( YRI )

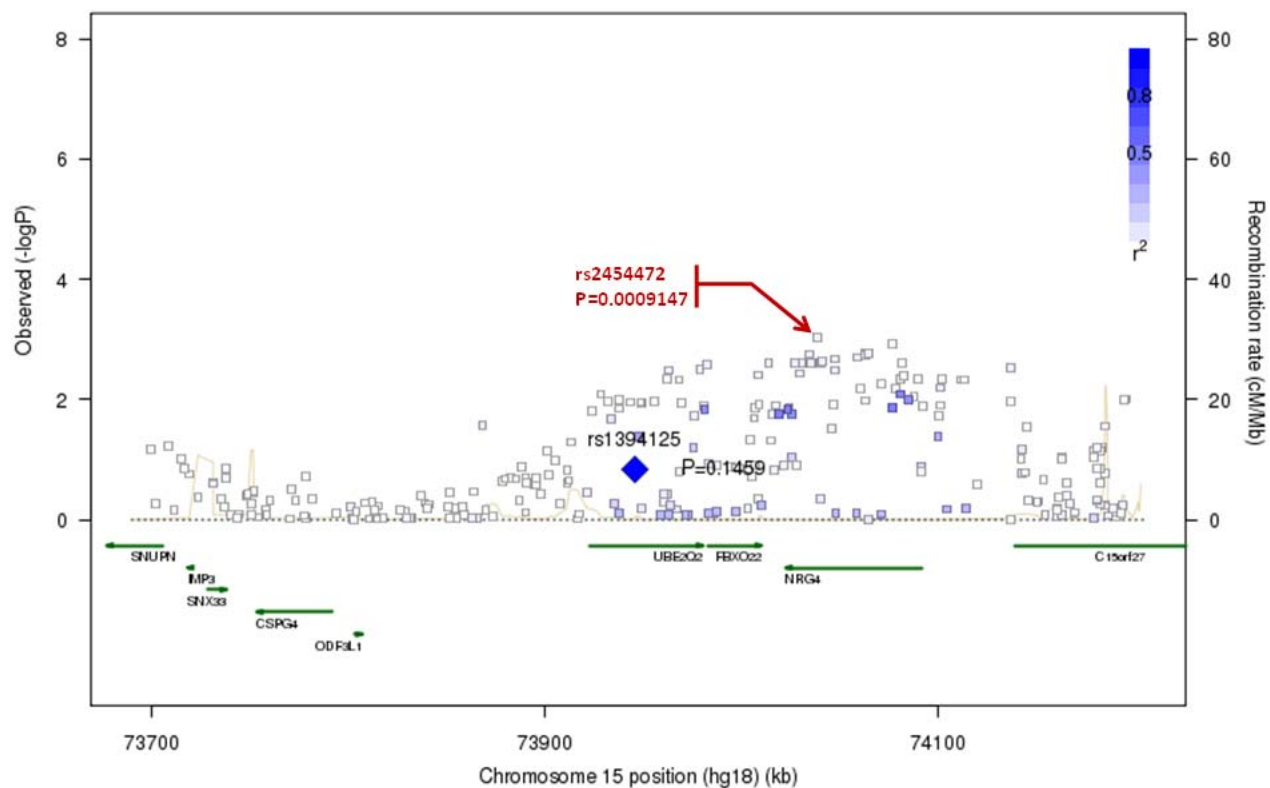

rs4293393 ( YRI )

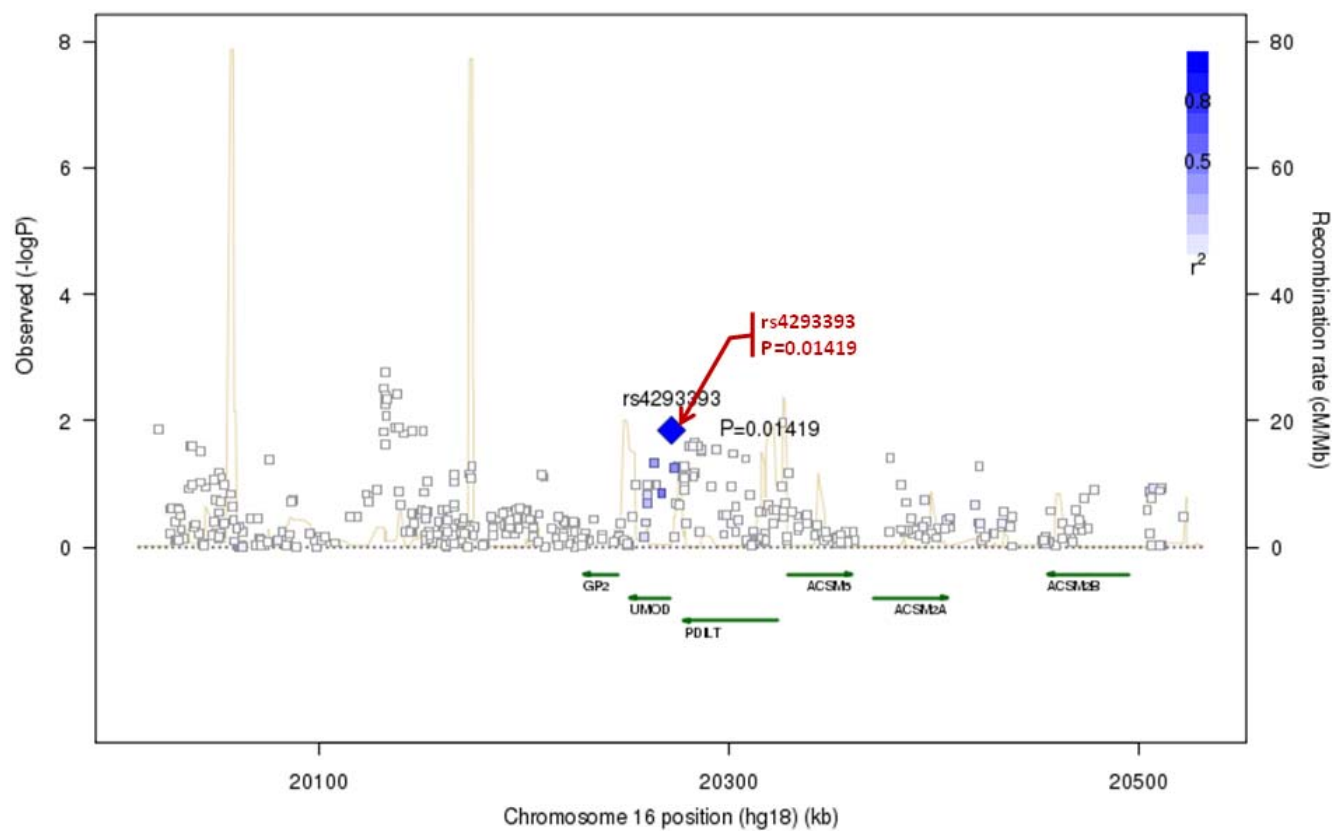

rs9895661 ( YRI )

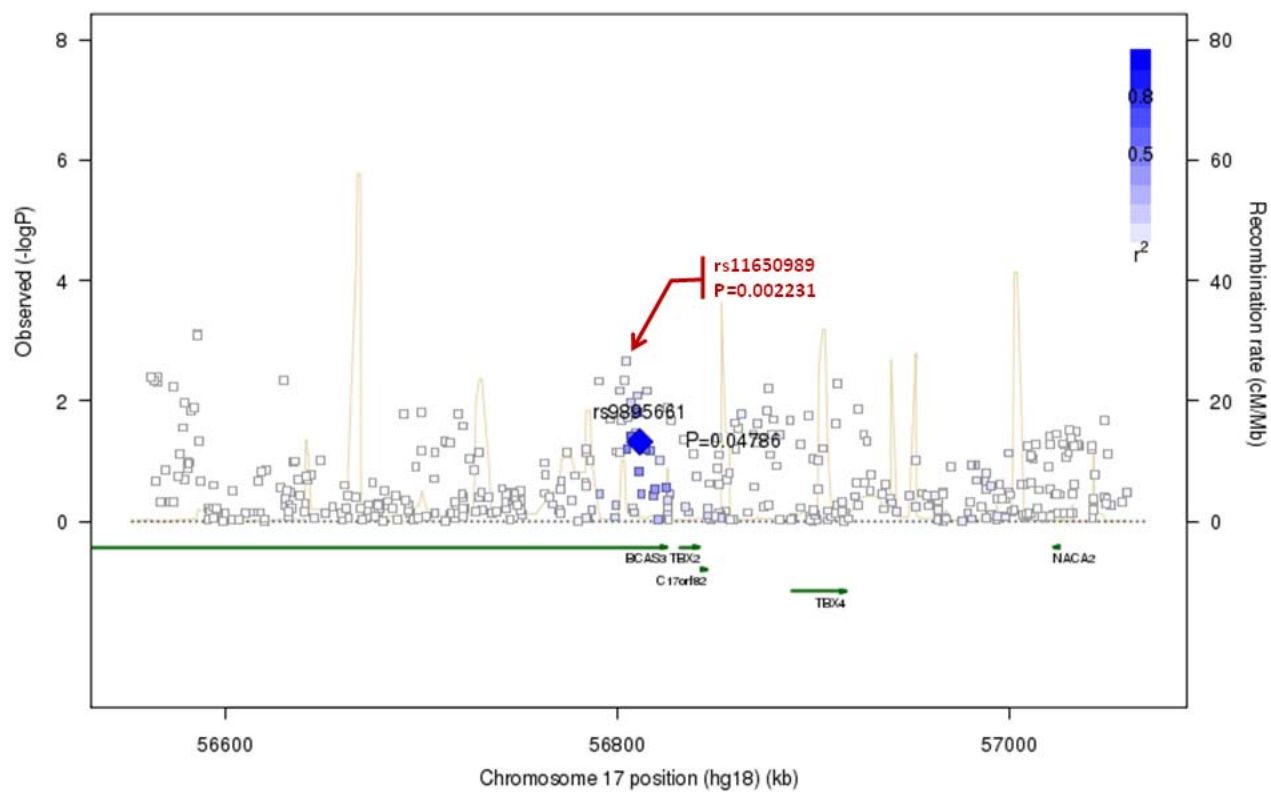

Supplement: Figure S3 — Regional association plots for all confirmed or replicating loci from the CKDGen loci interrogation; the blue notation represents the best SNP in whites with the p-value in African Americans, whereas red represents the lead SNP in African ancestry participants; the linkage disequilibrium shown uses YRI information from Hapmap2. (PDF) [file pgen.1002264.s003.pdf]

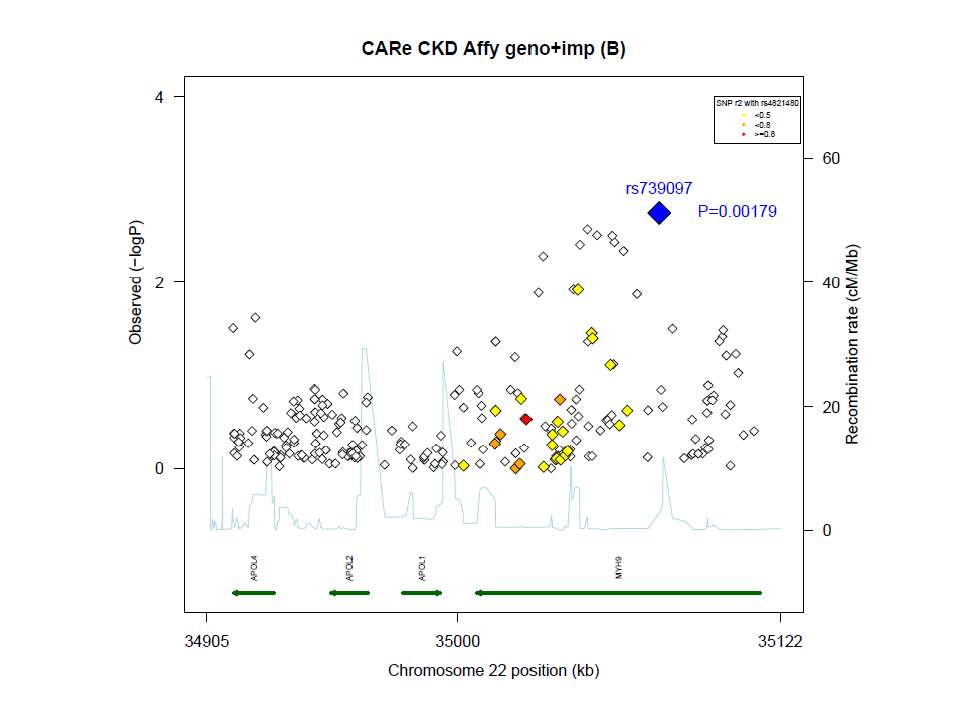

Supplement: Figure S4 — Regional association plot for the MYH9-APOL1 region in African ancestry participants. (TIF) [file pgen.1002264.s004.tif]

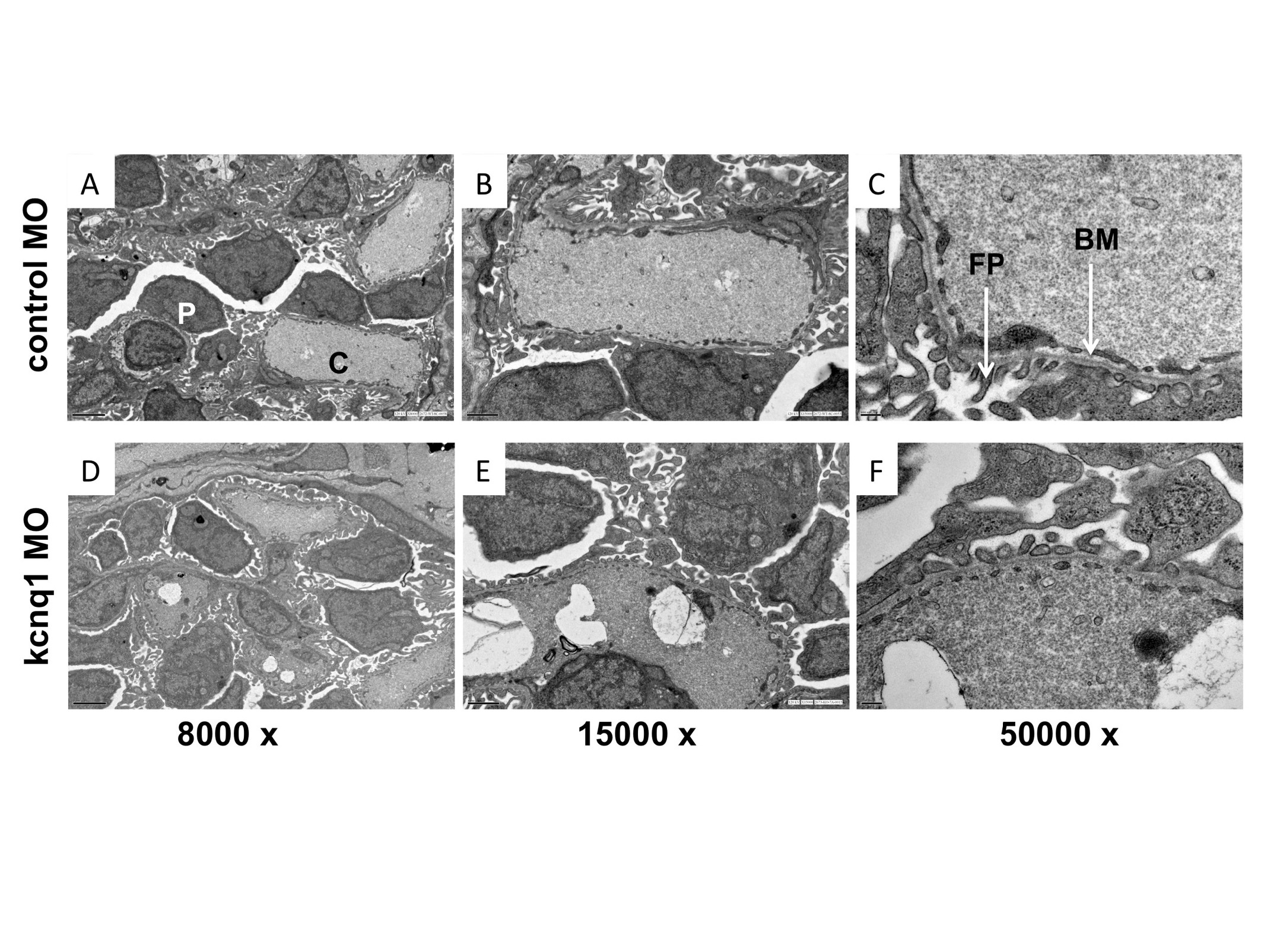

Supplement: Figure S5 — Analysis of glomerular architecture after kcnq1 knockdown by electron microscopy does not reveal significant changes. (a–c) Glomerular architecture at 120 hpf after injection of control morpholino visualized by electron microscopy at 8000-, 15,000, and 50,000-fold magnification reveals endothelial capillaries (C) with basement membrane (BM), podocytes (P) and foot processes (FP) similar to mammalian glomerular ultrastructure. (d–f) Transient knockdown of kcnq1 does not significantly change glomerular anatomy. (TIF) [file pgen.1002264.s005.tif]

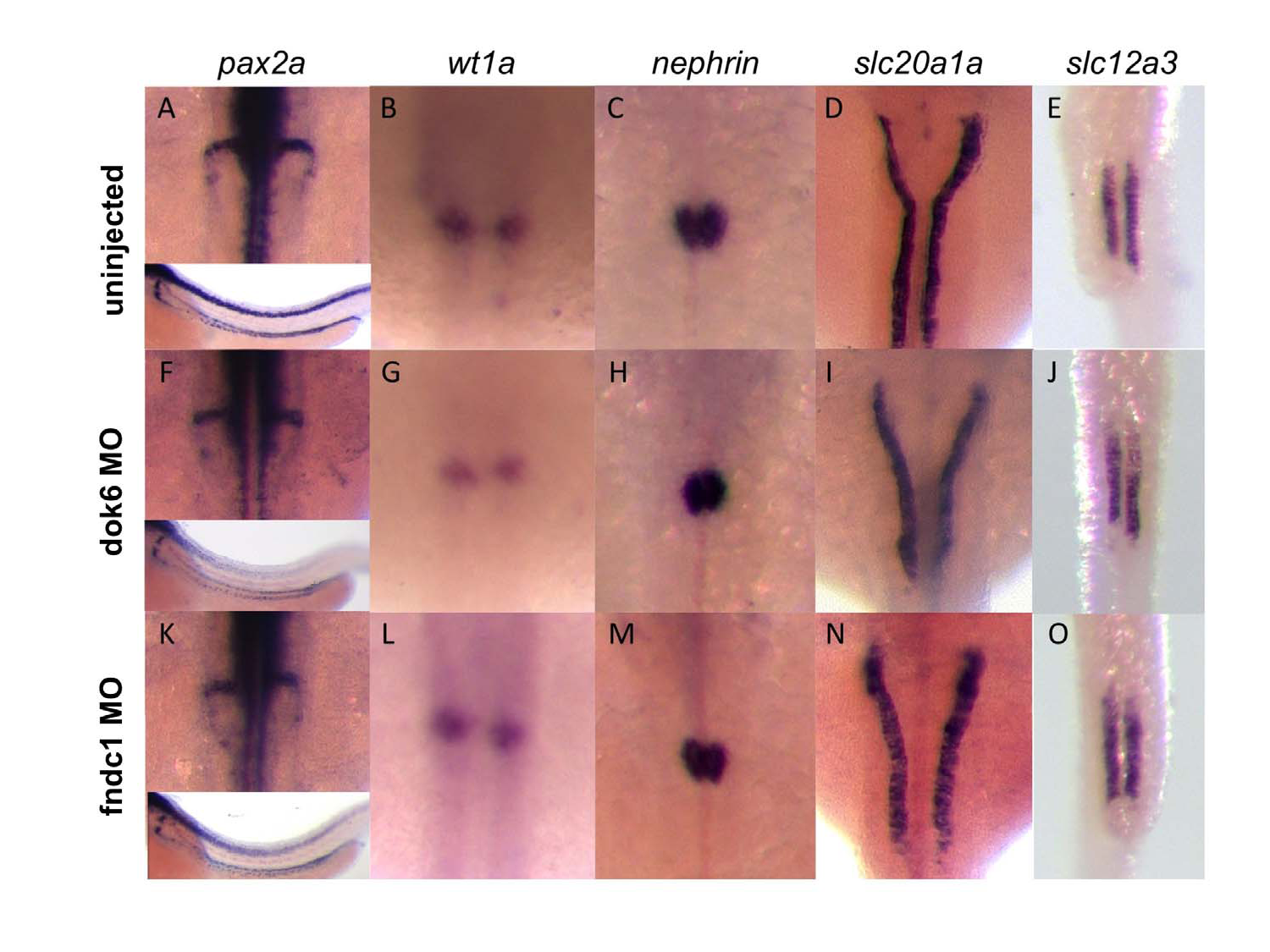

Supplement: Figure S6 — Dok6 and fndc1 knockdown in zebrafish embryos does not affect kidney development. (a–e) Uninjected control zebrafish embryos show normal glomerular and tubular morphology, as shown by in situ hybridization for the global kidney marker pax2a (a, inset showing lower-magnification image, with staining in both glomerulus and tubules), the podocyte markers wt1a (b) and nephrin (c), and the proximal and distal tubular markers slc20a1a (d) and slc12a3 (e). (f–o) Injection of dok6 (f–j) or fndc1 (k–o) morpholinos at the one-cell stage results in no significant changes in glomerular or tubular gene expression. (TIF) [file pgen.1002264.s006.tif]

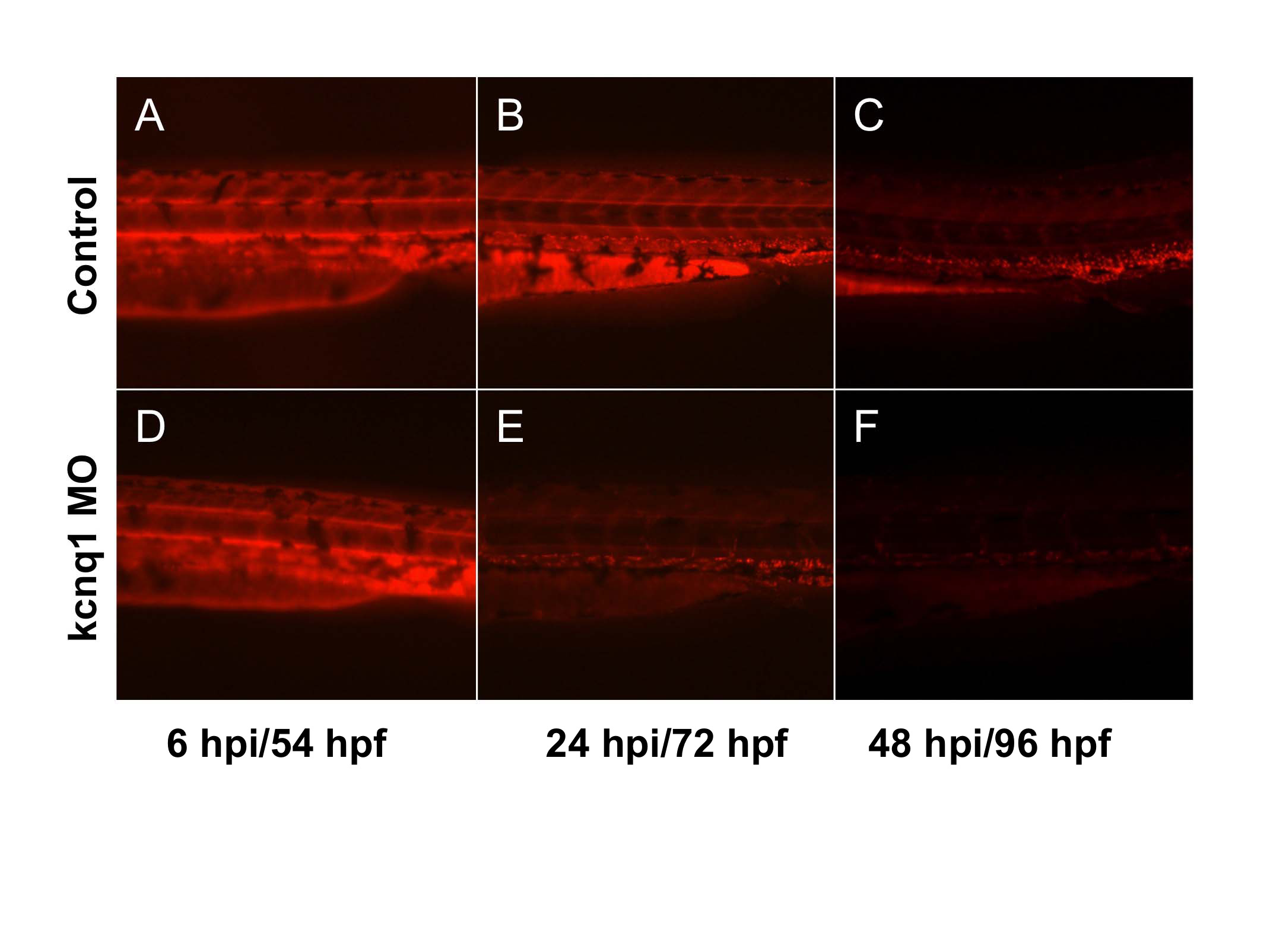

Supplement: Figure S7 — kcnq1 knockdown enhances loss of fluorescent dextran. Control and kcnq1 MO injected embryos were loaded with rhodamine-labeled dextran at 48 hpf. >80 embryos were analyzed for each group in 3 separate experiments (a,d) Fluorescence microscopy at 6 hpi reveals equal fluorescence loading between embryos. (b,e) At 72 hpf (24 hpi), fluorescence intensity in kcnq1 morphants is diminished. (c,f) At 96 hpf (48 hpi) control embryos retain their fluorescence, but kcnq1 morphants have significantly diminished fluorescence. (TIF) [file pgen.1002264.s007.tif]
