# Supplementary material for: Genetic Association for Renal Traits among Participants of African Ancestry Reveals New Loci for Renal Function
Source: PLoS Genet. 2011 Sep 8;7(9):e1002264. doi: 10.1371/journal.pgen.1002264 (PMC3169523; doi:10.1371/journal.pgen.1002264)
Supplement: Table S1 — Genotyping and imputation platforms. (DOC) [file pgen.1002264.s008.doc]

| Table S1- Genotyping and Imputation Platforms | | | | | | | | |
| --- | --- | --- | --- | --- | --- | --- | --- | --- |
|  | Array type | Genotype calling | QC filters for genotyped SNPs used for imputation | No of SNPs used for imputation | Imputation | Imputation Backbone for phased CEU haplotypes (NCBI build) | Filtering of imputed genotypes | Data management and statistical analysis |
| Stage 1: GWAS | | | | | | | | |
| CARE Consortium | Affymetrix 6.0 | Birdseed v1.33 | all chip QC + pi_hat 0.05 for rate step | 763537 to 846628 | MACH, 2 rounds | combined CEU+YRI reference panel | MAF 1%, rsq_hat 0.3 | plink –-dosage |
|  | IBC Chip | Birdseed v1.33 | all chip QC + pi_hat 0.05 for rate step | 44487 to 46438 | MACH, 2 rounds | CEU HapMap 2; combined CEU+YRI reference panel | MAF 1%, rsq_hat 0.4 | plink –-dosage; R-LME/GEE (for FHS) |
| Stage 2: Replication | | | | | | | | |
| GENOA | Affymetrix 6.0 | Birdseed v2 | call rate < 95%, pHWE <10E-6 |  | MACH v1.0.16 | HapMap release 22 (build 36) | none | PLINK; linear mixed models (R multic); GEE models (R gee glm); robust variance option to account for relatedness |
| HANDLS | Illumina 1M | Illumina Beadstudio | HWE p-value > 1e-7, missing by haplotype p-values > 1e-7, minor allele frequency > 0.01, and call rate > 95% | 907763 | MACH, 2 rounds | combined haplotype data for HapMap Phase 2 YRI and CEU samples that includes monomorphic SNPs in either of the two constituent populations (release 22, build 36) | MAF 1%, rsq_hat 0.3 | R,PLINK,RELPAIR,MACH2 |
| Health ABC | Illumina Human1M-Duo | Illumina BeadStudio | call rate < 97%, HWE p<10E-06, MAF<1% | 1,007,948 | MACH v1.0.16 | 1:1 mixture of the CEPH:Yoruban reference panel (release 22, build 36) | none | R |
| HUFS/Rotimi Cohort | Affymetrix Genome-Wide Human SNP Array 6.0 | Birdseed v2 | MAF 1%, SNP call rate 95%, sample call rate 95% | 842152 | MACH, 2 rounds | combined HapMap phase II+III, NCBI build 36 | MAF 1%, SNP call rate 90%, HWE 1e-3, genotypic concordance 95% | plink, R |
