# Supplementary material for: Genetic Association for Renal Traits among Participants of African Ancestry Reveals New Loci for Renal Function
Source: PLoS Genet. 2011 Sep 8;7(9):e1002264. doi: 10.1371/journal.pgen.1002264 (PMC3169523; doi:10.1371/journal.pgen.1002264)
Supplement: Table S2 — Genome-wide significant loci: SNP imputation quality* in Discovery and Replication cohorts. (DOC) [file pgen.1002264.s009.doc]

Table S2 - Genome-wide Significant Loci: SNP Imputation Quality* in Discovery and Replication Cohorts.

| SNP | ARIC | CARDIA | JHS | | CHS | MESA | GENOA | HABC | HANDLS | Rotimi | Median Imputation Quality |
| --- | --- | --- | --- | --- | --- | --- | --- | --- | --- | --- | --- |
| rs4371638 | 0.65 | 0.67 | 0.67 | | NA | 0.66 | 0.58 | 0.88 | 0.89 | 0.49 | 0.66 |
| rs192841 | 0.76 | 0.78 | 0.73 | | NA | 0.77 | 0.71 | 0.99 | 1.00 | 0.74 | 0.76 |
| rs4293393 | 0.87 | 0.86 | 1.00 | | NA | 1.00 | 0.87 | 1.00 | 1.00 | 1.00 | 1.00 |
| rs3738479 | 0.95 | 0.89 | 0.91 | | NA | 0.91 | 0.90 | 0.97 | 0.97 | 0.89 | 0.91 |
| rs13022873 | 0.98 | 0.95 | 0.94 | | NA | 0.98 | 0.98 | 0.99 | 0.98 | 0.9645** | 0.98 |
| rs7600291 | 0.92 | 1.01 | 0.96 | | NA | 0.95 | 0.89 | 0.95 | 0.96 | 0.90 | 0.95 |
| rs6781340 | 0.92 | 0.87 | 0.93 | | NA | 0.93 | 0.89 | 0.90** | 0.88** | 0.92 | 0.91 |
| rs3822460 | 1.00 | 1.00 | 1.00 | | NA | 1.00 | 0.98 | 0.95 | 0.94 | 1.00 | 1.00 |
| rs10463065 | 0.74 | 0.70 | 0.73 | | NA | 0.75 | 0.68 | 0.89 | 0.87 | 0.81 | 0.75 |
| rs1750571 | 1.00 | 1.00 | 1.00 | | NA | 1.00 | 0.97 | 0.92 | 0.78 | 1.00 | 1.00 |
| rs6464167 | 0.47 | 0.44 | 0.46 | | NA | 0.49 | 0.42 | 1.00 | 0.99 | 0.62 | 0.48 |
| rs1556751 | 1.01 | 1.02 | 0.97 | | NA | 0.98 | 0.99 | 1.00 | 0.99 | 0.96 | 0.99 |
| rs12302645 | 0.89 | 0.98 | 0.92 | | NA | 0.92 | 0.85 | 0.99 | 1.00 | 0.86 | 0.92 |
| rs9318029 | 0.62 | 0.64 | 0.61 | | NA | 0.66 | 0.62 | 0.93 | 0.92 | 0.84 | 0.65 |
| rs2454472 | 0.94 | 0.95 | 0.96 | | NA | 0.95 | 0.94 | 0.93 | 0.93 | 0.99** | 0.94 |
| rs3795058 | 0.95 | 0.92 | 0.93 | | NA | 0.95 | 0.92 | 0.94 | 0.92 | 0.89 | 0.93 |
| rs2805575 | 1.00 | 1.00 | 1.00 | | NA | 1.00 | 0.93 | 0.84 | 0.83 | 1.00 | 1.00 |
| rs16966247 | 0.89 | 0.91 | 0.84 | | NA | 0.86 | 0.91 | 0.90 | 0.90 | 0.98** | 0.90 |
| rs1153859 | 1.04 | 1.06 | 1.02 | | NA | 1.00 | 1.00 | 0.99 | 0.99 | 0.99** | 1.00 |
| rs957749 | 1.00 | 1.00 | 1.00 | | NA | 1.00 | 0.81 | 1.00 | 0.99 | 1.00 | 1.00 |
| rs3798156 | 0.90 | 0.86 | 0.90 | | NA | 0.90 | 0.87 | 0.91 | 0.88 | 0.82 | 0.89 |
| rs6973213 | 1.00 | 1.00 | 1.00 | | NA | 1.00 | 0.96 | 0.86 | 0.63 | 1.00 | 1.00 |
| rs485514 | 0.74 | 0.78 | 0.77 | | NA | 0.76 | 0.66 | 0.87 | 0.88 | 0.44 | 0.77 |
| rs11650989 | 1.00 | 1.00 | 1.00 | | NA | 1.00 | 1.00 | 1.00 | 0.99 | 1.00 | 1.00 |
| rs4555246 | 0.89 | 0.94 | 0.91 | | NA | 0.87 | 0.89 | 0.97 | 0.96 | *** | 0.91 |
| rs13213851 | 0.98 | 1.00 | 1.03 | | NA | 0.98 | 0.98 | 0.96 | 0.95 | *** | 0.98 |
| rs2880072 | 1.01 | 0.94 | 1.02 | | NA | 0.99 | 0.98 | 1.00 | 1.00 | *** | 1.00 |
| rs1009840 | 0.98 | 0.98 | 0.97 | | NA | 0.98 | 0.96 | 1.00 | 0.18 | *** | 0.98 |
| rs6581768 | 0.84 | 0.84 | 0.88 | | NA | 0.87 | 0.80 | 0.73** | 0.64** | 0.75 | 0.82 |
| rs7784820 | 1.00 | 0.97 | 0.98 | | NA | 0.94 | 0.96 | 0.91 | 0.91 | 0.97 | 0.96 |
| rs12575381 | 0.88 | 0.85 | 0.90 | | NA | 0.90 | 0.92 | 0.97 | 0.21 | 0.99** | 0.90 |
| rs6428106 | 0.95 | 0.87 | 0.89 | | NA | 0.93 | 0.91 | 0.91 | 0.84 | 0.97** | 0.91 |
| rs7111394 | 1.00 | 1.00 | 1.00 | | 1.00 | 1.00 | 0.82 | 0.98 | 0.99 | 0.71 | 1.00 |
| rs6865647 | NA | NA | NA | | 0.79 | NA | 0.78 | 0.99 | 0.99 | *** | 0.89 |
| rs4648006 | 1.00 | 1.00 | 1.00 | | 1.00 | 1.00 | 0.98 | 0.95 | 0.86 | 1.00 | 1.00 |
| *1.0 denotes a genotyped SNP  ** proxy | | | |  | | | | | | | |
| ***missing. No phenotype data | | | |  | | | | | | | |
