# Supplementary material for: Genetic Association for Renal Traits among Participants of African Ancestry Reveals New Loci for Renal Function
Source: PLoS Genet. 2011 Sep 8;7(9):e1002264. doi: 10.1371/journal.pgen.1002264 (PMC3169523; doi:10.1371/journal.pgen.1002264)
Supplement: Table S3 — Known loci for eGFRcrea and eGFRcys among participants of European Ancestry present on the IBC chip. (DOC) [file pgen.1002264.s010.doc]

Table S3 – Known loci for eGFRcrea and eGFRcys among participants of European Ancestry present on the IBC chip

| Trait | rsnumber | Chr | position (b36) | Genes In or Nearby | SNP function | Coded Allele Frequency | Beta | Stage 1 P-value |
| --- | --- | --- | --- | --- | --- | --- | --- | --- |
| eGFRcrea | rs9944411 | 17 | 34737125 | FBXL20 | intron | 0.74 (T) | -0.012 | 0.000000837 |
| eGFRcrea | rs6546837 | 2 | 73531406 | ALMS1 | coding exon | 0.24 C | 0.013 | 3.28E-08 |
| eGFRcrea* | rs12922822 | 16 | 20275146 | UMOD | promoter | 0.18 (T) | 0.014 | 6.48E-08 |
| eGFRcrea | rs2887915 | 2 | 211262819 | CPS1 | N/A | 0.33 (T) | 0.017 | 9.09E-08 |
| eGFRcrea | rs316029 | 6 | 160606658 | SLC22A2 | promoter | 0.14 (T) | 0.015 | 2.597E-07 |
| eGFRcrea | rs9369425 | 6 | 43918952 | VEGFA | N/A | 0.71 (A) | -0.011 | 1.18E-06 |
