# Supplementary material for: Genetic Association for Renal Traits among Participants of African Ancestry Reveals New Loci for Renal Function
Source: PLoS Genet. 2011 Sep 8;7(9):e1002264. doi: 10.1371/journal.pgen.1002264 (PMC3169523; doi:10.1371/journal.pgen.1002264)
Supplement: Table S4 — Cross-trait associations for novel loci from Stage 1+Stage 2 in participants of African ancestry. (DOC) [file pgen.1002264.s011.doc]

| **Table S4 - Cross-trait Associations for Novel Loci from Stage 1 + Stage 2 in Participants of African ancestry** | | | | | |
| --- | --- | --- | --- | --- | --- |
| **Trait** |  | **UACR** | **MA** | **eGFRcrea** | **CKD** |
| DOK6 | Beta or OR* | 0.1402 | 1.2 | -0.0053 | 0.99 |
| rs4555246 | SE or 95% CI | 0.028 | (1.07, 1.33) | 0.0044 | (0.86, 1.13) |
| Coded allele A | p-value | 5.33E-07 | 0.001 | 0.2303 | 0.9204 |
|  |  |  |  |  |  |
| FNDC1 | Beta or OR* | -0.1221 | 0.83 | 0.0006 | 1.04 |
| rs2880072 | SE or 95% CI | 0.0238 | (0.76, 0.91) | 0.0038 | (0.92, 1.17) |
| Coded allele A | p-value | 2.98E-07 | 4.90E-05 | 0.8647 | 0.46 |
|  |  |  |  |  |  |
| KCNQ1 | Beta or OR* | 0.0069 | 1.02 | -0.0213 | 1.07 |
| rs7111394 | SE or 95% CI | 0.0283 | (0.91, 1.13) | 0.0046 | ( 0.92, 1.22) |
| Coded allele T | p-value | 0.81 | 0.73 | 3.61E-06 | 0.36 |
| *beta for UACR and eGFRcrea and OR for MA and CKD | | |  |  |  |
