# Supplementary material for: Genetic Association for Renal Traits among Participants of African Ancestry Reveals New Loci for Renal Function
Source: PLoS Genet. 2011 Sep 8;7(9):e1002264. doi: 10.1371/journal.pgen.1002264 (PMC3169523; doi:10.1371/journal.pgen.1002264)
Supplement: Table S5 — Diabetes and Hypertension stratified analyses, Stage 1 data. (DOC) [file pgen.1002264.s012.doc]

Table S5 - Diabetes and Hypertension stratified analyses, Stage 1 data

|  |  |  | **DM (n=1385)** | |  | **Non-DM (6675)** | |  | **HTN (n=4039)** | |  | **Non-HTN (n=4048)** | | |
| --- | --- | --- | --- | --- | --- | --- | --- | --- | --- | --- | --- | --- | --- | --- |
| **trait** | **SNP ID** | **Genes In or Nearby** | **beta** | **SE** | **pvalue** | **beta** | **SE** | **pvalue** | **beta** | **SE** | **pvalue** | **beta** | **SE** | **pvalue** |
| eGFR | rs4293393 | UMOD | -0.007 | 0.0176 | 0.6901 | -0.0137 | 0.0055 | 0.01252 | -0.0201 | 0.009 | 0.0236 | -0.007 | 0.006 | 0.2529 |
| eGFR | rs3738479 | ANXA9 | 0.04 | 0.0151 | 0.0079 | 0.0087 | 0.0045 | 0.05271 | 0.0167 | 0.007 | 0.0207 | 0.0088 | 0.005 | 0.08315 |
| eGFR | rs13022873 | **GCKR** | 0.0098 | 0.0194 | 0.6121 | 0.0133 | 0.0054 | 0.01356 | 0.0137 | 0.009 | 0.1223 | 0.015 | 0.006 | 0.01341 |
| eGFR | rs6781340 | TFDP2 | -0.0351 | 0.0153 | 0.0216 | -0.0101 | 0.0045 | 0.0239 | -0.0143 | 0.007 | 0.0484 | -0.0119 | 0.005 | 0.01764 |
| eGFR | rs3822460 | **DAB2;**  C9 | -0.0087 | 0.0192 | 0.6487 | -0.013 | 0.0057 | 0.02227 | -0.0134 | 0.009 | 0.1446 | -0.0096 | 0.006 | 0.1338 |
| eGFR | rs1750571 | VEGFA | 0.0665 | 0.0271 | 0.014 | 0.018 | 0.008 | 0.02464 | 0.0337 | 0.013 | 0.0094 | 0.0117 | 0.009 | 0.1915 |
| eGFR | rs12302645 | **ATXN2** | -0.0322 | 0.0328 | 0.3258 | -0.0146 | 0.0094 | 0.1205 | -0.0317 | 0.015 | 0.0368 | -0.003 | 0.011 | 0.775 |
| eGFR | rs1153859 | **SPATA5L1** | -0.0134 | 0.0144 | 0.3532 | -0.0128 | 0.0043 | 0.0026 | -0.0085 | 0.007 | 0.2165 | -0.0184 | 0.005 | 0.000108 |
| eGFR | rs3798156 | **SLC22A2** | -0.0393 | 0.0234 | 0.0929 | -0.0254 | 0.0069 | 0.00023 | -0.0363 | 0.011 | 0.0014 | -0.0217 | 0.008 | 0.004118 |
| eGFR | rs6973213 | **TMEM60** | -0.0404 | 0.0174 | 0.0205 | -0.0168 | 0.0053 | 0.0016 | -0.0247 | 0.009 | 0.0035 | -0.0123 | 0.006 | 0.04275 |
| eGFR | rs485514 | **SLC6A13** | 0.0641 | 0.0298 | 0.0316 | 0.0086 | 0.0083 | 0.3055 | 0.0105 | 0.014 | 0.4359 | 0.022 | 0.01 | 0.02059 |
| eGFR | rs11650989 | **BCAS3** | 0.0318 | 0.0178 | 0.0745 | 0.0122 | 0.0054 | 0.02369 | 0.0167 | 0.009 | 0.058 | 0.0129 | 0.006 | 0.0305 |
| UACR | rs4555246 | **DOK6** | 0.1898 | 0.108 | 0.0788 | 0.1288 | 0.03 | 1.71E-05 | 0.1534 | 0.05 | 0.002 | 0.1399 | 0.034 | 3.08E-05 |
