# Supplementary material for: Genetic Association for Renal Traits among Participants of African Ancestry Reveals New Loci for Renal Function
Source: PLoS Genet. 2011 Sep 8;7(9):e1002264. doi: 10.1371/journal.pgen.1002264 (PMC3169523; doi:10.1371/journal.pgen.1002264)
Supplement: Table S6 — Morpholino sequences. (DOC) [file pgen.1002264.s013.doc]

**Table S6**

| Morpholino | Splice/ATG | Sequence (5’ – 3’) |
| --- | --- | --- |
| Kcnq1 | ATG | TTGAGGAGAGACTTTCACGCCTGAT |
| Kcnq1 | Splice | ATGAGGAATCTGCATAGGGAAAGAA |
| Dok6 | ATG | ATATCGTTGAAGTTTGAAGCCATCC |
| Fndc1 | Splice | TAACATTTTGTCAGACTCACCATCC |
