# Supplementary material for: Genetic Association for Renal Traits among Participants of African Ancestry Reveals New Loci for Renal Function
Source: PLoS Genet. 2011 Sep 8;7(9):e1002264. doi: 10.1371/journal.pgen.1002264 (PMC3169523; doi:10.1371/journal.pgen.1002264)
Supplement: Text S1 — Study-specific methods. (DOC) [file pgen.1002264.s014.doc]

**Study-Specific Methods**

**Discovery Cohorts**

**ARIC**: The ARIC study is a population-based, prospective study of clinical and subclinical atherosclerosis in four US communities: Forsyth County, North Carolina; Jackson, Mississippi; suburb of Minneapolis, Minnesota; and. Washington County, Maryland.  From 1987 to 1989, 15,792 white and African American participants aged 45–64 years were recruited by probability sampling and underwent baseline examination (visit 1).  Individuals were followed over time, which included three more clinical visits (visits 2-4), each scheduled approximately 3 years apart.  The analysis for eGFR using creatinine was performed using visit 1 data, and the analysis for UACR was performed using visit 4 data.  For this study, participants were excluded for non-consent to genetic research, or if they did not self-identify as black or white. Institutional Review Boards of the participating institutions (Johns Hopkins University, University of Minnesota, Wake Forest University, University of Mississipi, Baylor University, University of Texas and University of North Carolina) approved the study protocols. All participants provided written informed consent.

**CARDIA**: The study design details of the Coronary Artery Risk Development In Young Adults (CARDIA) have been published previously [1]. Briefly, CARDIA recruited a cohort of young white and black adults, ranging from 18-30 years at baseline. Between 1985-1986, participants were recruited from four sites: Birmingham, AL, Chicago, IL, Minneapolis, MN, and Oakland, CA. Subsequent examinations occurred at Years 2, 5, 7, 10, 15, and 20. Albuminuria was measured from a spot sample, and quantified by nephelometry. Creatinine was assessed using the Jaffe method. Urinary albumin and creatinine from Year 15 were reported as albumin to creatinine ratio in mg/g. Relevant covariates were measured at year 15 exam.

**CHS**: The Cardiovascular Health Study (CHS) is a longitudinal community-based study of risk factors for stroke and cardiovascular disease among adults aged 65 years or older. Recruitment occurred at four field centers (Forsyth County, NC; Sacramento County, CA; Washington County, MD; Pittsburgh, PA) [2]. Overall, 5201 predominantly white participants were recruited from 1989-1990 using random samples from Medicare eligibility lists. In 1992-1993, an additional 687 participants of African ancestry were recruited (n=5888). Using a single morning void, urine was collected; urinary albumin was measured by rate nephelometry using the Array 360 CE Protein Analyzer (Beckman Instruments, Fullerton, CA). Urine creatinine was measured using a Kodak Ektachem 700 Analyzer (Eastman Kodak company, Rochester, NY).

**FHS:**  The Framingham Heart Study began in 1948 with the enrollment of 5209 participants in the Original Cohort [3]. The Offspring Cohort was enrolled beginning in 1971 (5,124 participants), as has been previously described [4,5]. In 2002, the Third Generation cohort was enrolled (n=4095) [6]. Participants for the current study include individuals from the offspring cohort who attended the sixth exam (1995-1998, albuminuria), the seventh exam (1998-2001, eGFR and CKD), and individuals from the Third Generation first examination (2002-2005, eGFR and UACR). Serum creatinine was measured using the modified kinetic Jaffe reaction. On stored urine samples, urinary albumin concentration was measured using a Tina-quant immunoturbimetric assay (intra-assay CV 7.2% for the Offspring cohort, Third Generation CV= 2.1%; Roche Diagnostics, Indianapolis, Indiana). Using a modified Jaffe method, urinary creatinine concentration was measured (intra-assay CV=2.3% for the Offspring cohort and 1.0% for the Third Generation cohort).

**JHS**: The Jackson Heart Study is a single site longitudinal population-based study. The sample consists of 5,302 African-American women and men selected between 2000 and 2004 (first visit) from the tricounty area encompassing Jackson MS [7]. Spot urine collections were obtained; urine albumin and creatinine concentration were measured with the human albumin kit (Dade, Behring,Newark ,DE) on the Dade Behring BN II nephelometer. Biochemical testing for serum and creatinine was performed at the University of Mississippi Medical Center Laboratory Reading Center with a multipoint enzymatic spectrophotometric assay (Vitros CREA dry reaction slides on a Vitros 950 Otho-Clinical Diagnositics analyzer, Raritan, NJ). Creatinine values were biochemically calibrated to the Cleveland Clinic-equivalent Minnesota Beckman CX3 assay (Beckman-Coulter Inc, Fullteron, CA).

**MESA** The Multi-Ethnic Study of Atherosclerosis (MESA) is a community-based cohort study designed to study the prevalence, correlates, and progression of subclinical cardiovascular disease [8]. Overall, MESA consists of 6,814 adults of diverse race/ethnicity (38% white, 28% African ancestry, 22% Hispanic, and 12% Chinese) from the following regions: Forsyth County, North Carolina, Northern Manhattan and the Bronx, New York, Baltimore City and Baltimore County, Maryland, St. Paul, Minnesota, Chicago, Illinois, and Los Angeles County, California. Participants were between the ages of 45 to 84 years and were free of cardiovascular disease at enrollment (2000-2002). All data used for the analyses reported herein were collected at this baseline examination. Serum creatinine was measured by rate reflectance spectrophotometry using thin film adaptation of the creatine amidinohydrolase method on the Vitros analyzer (Johnson & Johnson Clinical Diagnostics, Inc., Rochester, NY 14650) at the Collaborative Studies Clinical Laboratory at Fairview-University Medical Center (Minneapolis, MN) and calibrated to Cleveland Clinic. Urine was collected from single voided specimens; urine albumin concentration was measured by nephelometry using the Array 360 CE Protein Analyzer (Beckman Instruments, Inc., Drea, CA). Urine creatinine was measured using the Vitros 950IRC instrument (Johnson & Johnson Clinical Diagnostics, Inc., Rochester, NY) with a CV range of 2.5 – 2.9%.

**Replication Cohorts**

**Howard University Family Study Methods**  The Howard University Family Study is a population-based study of African American families enrolled from the Washington, D.C. metropolitan area [9]. In the first phase of recruitment, a randomly ascertained cohort of 350 African American families with members in multiple generations from the Washington, D.C. metropolitan area were enrolled and examined. In a second phase of recruitment, additional unrelated individuals from the same geographic area were enrolled to facilitate nested case-control study designs. The total number of recruited individuals was 2,028, of which 1,976 remained after data cleaning. From this sample, we created a subset of 1,055 unrelated individuals. Serum creatinine was measured on fasting morning samples using the modified Jaffe method. Evidence for population stratification was obtained through nonparametric clustering of genotypes using the R package AWclust [10]. Two-dimensional projections from principal coordinate analysis were drawn using R. Individual admixture proportions were estimated using a panel of 2,076 ancestry-informative markers (AIMs) assuming two populations and uncorrelated allele frequencies with a 10,000 step burn-in and a 1,000 step chain using STRUCTURE 2.2 [11]. We performed linear regression for eGFR and logistic regression for CKD using the glm function in R.

**GENOA** The Family Blood Pressure Program (FBPP), established by the National Heart Lung and Blood Institute in 1996, joined existing research networks that were investigating hypertension and cardiovascular diseases (<http://public.nhlbi.nih.gov/GeneticsGenomics/home/fbpp.aspx>). One of the four FBPP networks is the Genetic Epidemiology Network of Arteriopathy (GENOA), which recruited hypertensive, Caucasian and African American sibships for linkage and association studies to investigate genetic contributions to hypertension and hypertension-related target organ damage. Serum creatinine was measured using a modified kinetic Jaffé reaction. Using stored urine samples, urine albumin and creatinine concentrations were measured by standard method on a Hitachi 911 Chemistry Analyzer (Roche Diagnostics, Indianapolis, IN) for GENOA participants.

**Health ABC Study** The Health ABC Study is a community-based prospective cohort study that began in 1997, consisting of 1200 African-American men and women in the original cohort. Stored urine was assayed by Synarc Inc., Paris, France. Urinary albumin concentration was measured with a nephelometric method using a Nephelometer-Analyzer (intra-assay CV 17.8%; BEHRING BNA, SIEMENS). Urinary creatinine concentration was measured using a modified Jaffe method (Colorimetric method) on a KONE 20 analyzer (intra-assay CV 15.4%; KONELAB). Serum creatinine was measured using a modified kinetic Jaffé reaction; serum cystatin C was measured using particle-enhanced immunonephelometric method (BNII, Dade-Behring). Genomic DNA was extracted from buffy coat collected using PUREGENE® DNA Purification Kit during the baseline exam. In 2009, genotyping was performed by the Center for Inherited Disease Research (CIDR).

**The Healthy Aging in Neighborhoods of Diversity across the Life Span Study (HANDLS)** The Healthy Aging in Neighborhoods of Diversity across the Life Span study (HANDLS) is a community-based, prospective longitudinal epidemiologic study among socioeconomically diverse African Americans and whites in Baltimore. The HANDLS design is an area probability sample of Baltimore based on the 2000 Census. Genotyping was focused on a subset of participants self-reporting as African American; overall, 1024 participants were successfully genotyped to 907763 SNPS at the equivalent of Illumina 1M SNP coverage (709 samples using Illumina 1M and 1Mduo arrays, the remainder using a combination of 550K, 370K, 510S and 240S to equate the million SNP level of coverage). Ancestry estimates were assessed using both STRUCTUREv2.3 [11–13] and the multidimensional scaling (MDS) function in PLINKv1.06. HANDLS participant genotypes were imputed using MACHv1.0.16 (<http://www.sph.umich.edu/csg/abecasis/mach/>) based on combined haplotype data for HapMap Phase 2 YRI and CEU samples. Blood albumin and creatinine measures were taken from the same blood draw using spectrophotometric methods for both. Urine albumin concentration and creatinine concentrations were measured using turbidometric methodology.

Reference List

1. Friedman GD, Cutter GR, Donahue RP, Hughes GH, Hulley SB et al. (1988) CARDIA: study design, recruitment, and some characteristics of the examined subjects. J Clin Epidemiol 41: 1105-1116.

2. Fried LP, Borhani NO, Enright P, Furberg CD, Gardin JM et al. (1991) The Cardiovascular Health Study: design and rationale. Ann Epidemiol 1: 263-276.

3. Dawber T.R., Kannel W.B., Lyell L.P. (1963) An approach to longitudinal studies in a community: the Framingham Heart Study. Ann NY Acad Sci 107: 539-556.

4. Feinleib M, Kannel WB, Garrison RJ, McNamara PM, Castelli WP (1975) The Framingham Offspring Study. Design and preliminary data. Prev Med 4: 518-525.

5. Garrison RJ, Castelli WP, Feinleib M, Kannel WB, Havlik RJ et al. (1979) The association of total cholesterol, triglycerides and plasma lipoprotein cholesterol levels in first degree relatives and spouse pairs. Am J Epidemiol 110: 313-321.

6. Splansky GL, Corey D, Yang Q, Atwood LD, Cupples LA et al. (2007) The Third Generation Cohort of the National Heart, Lung, and Blood Institute's Framingham Heart Study: design, recruitment, and initial examination. Am J Epidemiol 165: 1328-1335.

7. Taylor HA, Jr. (2005) The Jackson Heart Study: an overview. Ethn Dis 15: S6-3.

8. Bild DE, Bluemke DA, Burke GL, Detrano R, ez Roux AV et al. (2002) Multi-ethnic study of atherosclerosis: objectives and design. Am J Epidemiol 156: 871-881.

9. Adeyemo A, Gerry N, Chen G, Herbert A, Doumatey A et al. (2009) A genome-wide association study of hypertension and blood pressure in African Americans. PLoS Genet 5: e1000564.

10. Gao X, Starmer JD (2008) AWclust: point-and-click software for non-parametric population structure analysis. BMC Bioinformatics 9: 77.

11. Falush D, Stephens M, Pritchard JK (2003) Inference of population structure using multilocus genotype data: linked loci and correlated allele frequencies. Genetics 164: 1567-1587.

12. Pritchard JK, Stephens M, Donnelly P (2000) Inference of population structure using multilocus genotype data. Genetics 155: 945-959.

13. Falush D, Stephens M, Pritchard JK (2007) Inference of population structure using multilocus genotype data: dominant markers and null alleles. Mol Ecol Notes 7: 574-578.
